# Refining short-range order parameters from the three-dimensional diffuse scattering in single-crystal electron diffraction data

**DOI:** 10.1107/S2052252523010254

**Published:** 2024-01-01

**Authors:** Romy Poppe, Nikolaj Roth, Reinhard B. Neder, Lukas Palatinus, Bo Brummerstedt Iversen, Joke Hadermann

**Affiliations:** a University of Antwerp, Department of Physics, Groenenborgerlaan 171, B-2020 Antwerp, Belgium; b University of Oxford, Inorganic Chemistry Laboratory, South Parks Road, Oxford OX1 3QR, United Kingdom; c Friedrich-Alexander-Universität Erlangen-Nürnberg, Kirstallographie und Strukturphysik, Staudtstraße 3, 91058 Erlangen, Germany; d Czech Academy of Sciences, Department of Structure Analysis, Na Slovance 2, 182 21 Prague, Czechia; e Aarhus University, Department of Chemistry and iNANO, Langelandsgade 140, 8000 Aarhus, Denmark; Ben-Gurion University of the Negev, Israel

**Keywords:** 3D electron diffraction, 3DED, single-crystal diffuse scattering, 3D difference pair distribution functions, 3D-ΔPDF

## Abstract

Our study compares short-range order parameters refined from the diffuse scattering in single-crystal X-ray and single-crystal electron diffraction data. Nb_0.84_CoSb was chosen as a reference material. The correlations between neighbouring vacancies and the displacements of Sb and Co atoms were refined from the diffuse scattering using a Monte Carlo refinement in *DISCUS*. The difference between the Sb and Co displacements refined from the diffuse scattering and the Sb and Co displacements refined from the Bragg reflections in single-crystal X-ray diffraction data is 0.012 (7) Å for the refinement on diffuse scattering in single-crystal X-ray diffraction data and 0.03 (2) Å for the refinement on the diffuse scattering in single-crystal electron diffraction data. As electron diffraction requires much smaller crystals than X-ray diffraction, this opens up the possibility of refining short-range order parameters in many technologically relevant materials for which no crystals large enough for single-crystal X-ray diffraction are available.

## Abstract 2

This study compares, for the first time, short-range order parameters refined from the diffuse scattering in single-crystal X-ray and single-crystal electron diffraction data.

## 1. Introduction

Structure solution and refinement are used to solve and refine the average structure of crystalline materials from the intensities of the Bragg reflections. Parameters that are most commonly refined are the average atomic positions, site occupancies and atomic displacement parameters. However, for many materials, the local structure differs significantly from the average structure. When the deviations from the average structure are ordered on a local scale, they are referred to as local order or short-range order. Materials with short-range order have diffraction patterns that contain both Bragg reflections and structured diffuse scattering. Because the properties of many materials depend on the short-range order, refining short-range order parameters is essential for understanding and optimizing material properties.

The development of three-dimensional electron diffraction (3DED) in 2007 (Kolb *et al.*, 2007 ▸, 2008 ▸) allowed the acquisition of electron diffraction data with less multiple scattering compared with in-zone electron diffraction patterns. The main advantage of 3DED is that it allows us to determine the crystal structure of materials for which no crystals large enough for single-crystal X-ray diffraction are available (Gemmi *et al.*, 2019 ▸). When refining the average structure from single-crystal X-ray diffraction data using a standard least-squares refinement, it is assumed that the intensities of the Bragg reflections are approximately proportional to the square of the absolute value of the structure factor. Because electrons are scattered multiple times while going through the crystal, this assumption is not valid for 3DED data. A least-squares refinement in which the intensities are calculated using the dynamical theory of diffraction was developed by Palatinus and co-workers (Palatinus *et al.*, 2013 ▸; Palatinus, Corrêa *et al.*, 2015 ▸; Palatinus, Petříček *et al.*, 2015 ▸) and is implemented in *Jana2020* (Petříček *et al.*, 2023 ▸).

**Figure 1:**
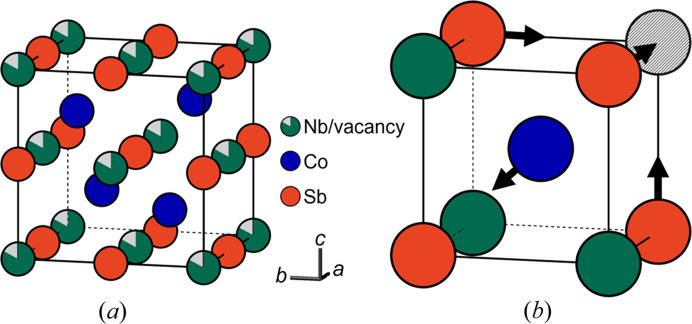
(*a*) Unit cell of the average crystal structure of Nb_0.84_CoSb. Nb and Sb atoms form a rock salt structure, whereas Nb and Co atoms form a sphalerite structure. (*b*) Arrows indicate the local displacements of Sb and Co atoms. Sb atoms move towards neighbouring vacancies, whereas Co atoms move away from neighbouring vacancies. Figure adapted from Roth *et al.* (2021 ▸).

In most studies on the diffuse scattering in single-crystal electron diffraction data, the diffuse scattering in experimental data is qualitatively compared with the diffuse scattering in calculated data (Withers *et al.*, 2003 ▸, 2004 ▸; Fujii *et al.*, 2007 ▸; Goodwin *et al.*, 2007 ▸; Brázda *et al.*, 2016 ▸; Zhao *et al.*, 2017 ▸; Neagu & Tai, 2017 ▸; Gorelik *et al.*, 2023 ▸). A quantitative analysis of the diffuse scattering in 3DED data has only been reported in the case of 1D diffuse scattering (Krysiak *et al.*, 2018 ▸, 2020 ▸; Poppe *et al.*, 2022 ▸). In this article, we refine short-range order parameters from the 3D diffuse scattering in 3DED data. The defective half-Heusler system Nb_0.84_CoSb (Fig. 1 ▸), previously studied using single-crystal X-ray diffraction (Roth *et al.*, 2021 ▸), was chosen as a reference material. The correlations between neighbouring vacancies and the displacements of the Sb and Co atoms were refined from the diffuse scattering using a Monte Carlo refinement in *DISCUS*. The Nb occupancy and the displacements of Sb and Co atoms were also refined from the Bragg reflections in 3DED data.

## 2. Experimental

### 2.1. Synthesis

The samples used in this study were previously used by Roth *et al.* (2021 ▸) and are referred to as ‘SC-0.81’ and ‘Q-0.84 #2’. Two different synthesis methods were used to prepare these samples. The SC-0.81 sample has nominal stoichiometry Nb_0.81_CoSb and was slowly cooled (SC) using an induction furnace. The Q-0.84 #2 sample has nominal stoichiometry Nb_0.84_CoSb and was thermally quenched (Q) from the melt. Details of the synthesis can be found in Roth *et al.* (2021 ▸) for the slowly cooled sample and in Yu *et al.* (2018 ▸) for the thermally quenched sample. The thermally quenched sample Nb_0.84_CoSb (Q-0.84 #2) has only short-range Nb-vacancy order, whereas the slowly cooled sample Nb_0.81_CoSb (SC-0.81) also has long-range Nb-vacancy order.

### 2.2. 3DED data collection

Samples for 3DED data collection were prepared by dispersing the powder in ethanol. A few droplets of the suspension were deposited on a copper grid covered with an amorphous carbon film. Ultra-thin amorphous carbon grids were used to reduce the experimental background.

3DED data were acquired with an aberration-corrected cubed FEI Titan 80–300 electron microscope operated at 300 kV using a GATAN US1000XP CCD camera (4096 × 4096 pixels with 16-bit dynamic range). The crystal was illuminated in selected area electron diffraction (SAED) mode with an exposure time of 1 s per frame. Electron diffraction patterns were acquired with a Fischione tomography holder (tilt range ±80°) in a stepwise manner using an in-house developed script.

The 3DED data used for the dynamical refinement were collected with a step size of 0.1° on crystals 80–200 nm in size. The crystals were entirely illuminated during the whole data collection. The 3DED data used for the diffuse scattering analysis were collected with a step size of 0.1 or 0.2° on crystals 200–3000 nm in size. For larger crystals, only a thin part of the crystal was illuminated, which was recentred inside the aperture every few degrees.

Because the Bragg reflections are three orders of magnitude stronger than the diffuse scattering, the acquisition of high-quality diffuse scattering data requires careful subtraction of the experimental background. *PETS2* (Palatinus *et al.*, 2019 ▸) was used to process the 3DED data including background subtraction of the individual frames, integration of the Bragg reflection intensities for the dynamical refinement and applying symmetry with Laue class *m* 3 *m* in the reconstruction of the 3D reciprocal lattice. Pixels with negative intensities were used as negative values. The 3D reciprocal lattice of all 3DED data was indexed with a cubic unit cell with the cell parameter *a* = 5.89864 (3) Å and space group *F* 4 *3m* (Zeier *et al.*, 2017 ▸).

Details on the acquisition of the single-crystal X-ray diffraction data can be found in Roth *et al.* (2021 ▸).

### 2.3. Dynamical refinement of the average crystal structure

Dynamical refinements of the average crystal structure were performed in *Jana2020*. Because the space group *F* 4 *3m* is non-centrosymmetric, a twinning inversion matrix was defined. Refined parameters include the Nb occupancy; the twin fraction or Flack parameter; a thickness parameter; harmonic displacement parameters of Sb, Nb and Co; and one scale factor per virtual frame. The Nb occupancy was allowed to refine freely, whereas the occupancies of Sb and Co were fixed to 1. The intensities in the dynamical refinement were calculated for a wedge-shaped crystal (Palatinus, Petříček *et al.*, 2015 ▸). The dynamical refinement parameters were set to *g*_max_ = 2.4 Å^−1^, *S*_g_^max^(matrix) = 0.025 Å^−1^, *S*_g_^max^(refine) = 0.1, 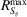 = 0.66 and *N*_int_ = 100. The meaning of the dynamical refinement parameters can be found in the supporting information.

### 2.4. Monte Carlo refinement

*DISCUS* (Proffen & Neder, 1997 ▸) was used to build a model of the short-range Nb-vacancy order in the thermally quenched sample Nb_0.84_CoSb (Q-0.84 #2) and of the long-range Nb-vacancy order in the slowly cooled sample Nb_0.81_CoSb (SC-0.81). Both models can be found in Poppe (2023*a* ▸).

The intensities in reciprocal space were calculated according to the standard formula for kinematic scattering 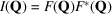 (Neder & Proffen, 2008 ▸). The structure factor *F*(**Q**) was calculated using the discrete Fourier transform (DFT):

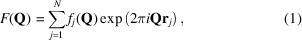

where *N* is the number of atoms in the crystal, *f_j_* (**Q**) is the atomic form factor of atom *j*, 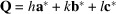 is a reciprocal lattice vector and 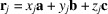 is the direct lattice vector of atom *j*.

Correlation coefficients 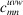 between neighbouring vacancies in *DISCUS* are defined as (Welberry, 1985 ▸; Neder & Proffen, 2008 ▸)

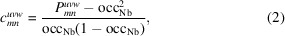

where 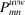 is the probability that sites *m* and *n* are occupied by the same atom type, *uvw* the interatomic vector, and 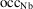 is the Nb occupancy. Negative values of 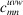 indicate that sites *m* and *n* tend to be occupied by different atom types whereas positive values of 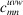 indicate that sites *m* and *n* tend to be occupied by the same atom type. A correlation value of zero describes a random distribution of the two atom types. The maximum negative value of 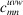 for a given occupancy 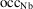 is 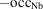 /(1 − 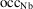 ) ( 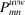 = 0), the maximum positive value is +1( 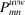 = 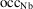 ).

The Monte Carlo refinement was applied to the diffuse scattering in the *h*0*l* plane from both single-crystal X-ray diffraction data and 3DED data acquired on the thermally quenched sample (Q-0.84 #2). The 3D X-ray and electron diffuse scattering data are available in Poppe (2023*d* ▸). The Bragg reflections were subtracted using *MANTID* (Arnold *et al.*, 2014 ▸), and the 3D diffuse scattering data were cropped on a grid with 215 × 215 pixels for −6 ≤ *h*,*l* ≤ 6. The intensities in the *h*0*l* plane were converted to input for *DISCUS* using a custom Python script (Poppe, 2023*c* ▸).

The model of the short-range Nb-vacancy order was implemented in a differential evolutionary algorithm. Three parameters were refined: the correlation between next-nearest neighbour vacancies and the displacements of Sb and Co atoms. A more detailed explanation of the *DISCUS* models and the Monte Carlo refinement is given in the supporting information. The Monte Carlo refinement (Poppe, 2023*b* ▸) took about seven days for 19 refinement cycles on a desktop computer using eight cores in parallel. The 24 children were calculated in parallel, whereas the individual crystals and the individual lots were calculated in series.

### 2.5. The 3D-ΔPDF

The 3D difference pair distribution function (3D-ΔPDF) was calculated in *MANTID*. *MANTID* was installed on a high-performance computing (HPC) cluster, and the 3D-ΔPDF was calculated in parallel on a node with 128 GB RAM. The Bragg reflections were subtracted, and the 3D-ΔPDF was obtained by Fourier transform of the 3D diffuse scattering/satellite reflections.

For the simulated 3D-ΔPDF maps, *Scatty* (Paddison, 2019 ▸) was used to calculate the 3D diffuse scattering from the *DISCUS* models. *Scatty* uses a fast Fourier transform (FFT) algorithm to calculate the structure factor in equation (1), which accelerates the calculation of the diffuse scattering by a factor 10^2^–10^3^ compared with the discrete Fourier transform (DFT). Lanczos resampling was used to reduce the high-frequency noise. For the short-range Nb-vacancy order model, the calculation of the 3D diffuse scattering took about eight days. The diffuse scattering was calculated for expansion order 10, expansion maximum error 0.05 and window 2. To obtain an error smaller than 5%, the outer part of the 3D reciprocal lattice (about 18%) was calculated using DFT. For the long-range Nb-vacancy order model, calculation of the 3D diffuse scattering took only 35 min. The diffuse scattering was calculated for expansion order 1 and window 2.

For the experimental 3D diffuse scattering data, the 3D reciprocal lattice was reconstructed on a grid with 901 × 901 × 901 voxels for −25.2 ≤ *h*,*k*,*l* ≤ 25.2. For the 3D diffuse scattering data calculated in *Scatty*, the 3D reciprocal lattice was reconstructed on a grid with 401 × 401 × 401 voxels for −20 ≤ *h,k,l* ≤ 20.

## 3. Results and discussion

### 3.1. Dynamical refinement of the average crystal structure

The percentage of vacancies on the Nb sites and the average displacements of Sb and Co atoms in the thermally quenched sample (Q-0.84 #2) were refined from the Bragg reflections in the 3DED data using a dynamical refinement, and were compared with reference values refined from the Bragg reflections in single-crystal X-ray diffraction data (Roth *et al.*, 2021 ▸).

3DED data were acquired on three different crystals, and each dataset was processed separately using *PETS2* (Palatinus *et al.*, 2019 ▸). Analogous to the refinement of the average structure from the Bragg reflections in single-crystal X-ray diffraction data (Roth *et al.*, 2021 ▸), the dynamical refinement of the average structure was done in two stages. In the first stage (centre model), the displacements of Sb and Co atoms were fixed to zero. In the second stage (split model), Sb atoms were off-centred at (1/2 + Δ, 1/2, 1/2), Co atoms were off-centred at (1/4 − δ, 1/4 − δ, 3/4 − δ), and the displacements of Sb and Co atoms (Δ and δ) were allowed to refine freely.

Results of the dynamical refinement for the centre model and for the split model are shown in Table 1 ▸. The refined atomic displacement parameters for Sb, Co and Nb are listed in Table S1 of the supporting information. The standard uncertainties on the Nb occupancy and the average displacements of Sb and Co atoms only consider random errors in the intensities of the Bragg reflections and are thus underestimated. Differences between the refined Nb occupancy and the refined Sb and Co displacements for the three crystals can be due to real differences in the Nb occupancy and the Sb and Co displacements between the three crystals or can be due to systematic errors in the calculation of the intensities in the dynamical refinement. Systematic errors can be attributed to (i) strong multiple scattering caused by the high atomic numbers of Nb, Sb and Co; (ii) the relatively low data-to-parameter ratio (*i.e.* the number of observed reflections per refined parameter); (iii) a high crystal mosaicity; and (vi) no optimization of the frame orientation angles. The orientations of the frames could not be optimized due to the limited number of reflections on each frame. The limited accuracy of the goniometer of the TEM stage or small unpredictable movements of the crystal during the acquisition of the data may cause the orientation of a frame, as calculated from the orientation matrix, to be inaccurate (Palatinus, Corrêa *et al.*, 2015 ▸). Note that the *R* values and the refined parameters have a slight dependence on the dynamical refinement parameters. For example, changing the value for 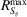 from 0.66 to 0.8 changed the refined Nb occupancy by 1.9% for crystal 1, by 0.3% for crystal 2 and by 0.1% for crystal 3.

The average Nb occupancy of the three crystals (x) was calculated using equation (3):

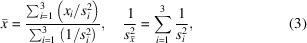

where *x_i_* is the Nb occupancy of each crystal and *s_i_* is the standard uncertainty on the Nb occupancy. For the split model, the average Nb occupancy refined from the Bragg reflections in 3DED data [0.813 (11)] differs by only 0.014 (11) from the Nb occupancy refined from the Bragg reflections in single-crystal X-ray diffraction data [0.827 (2)]. Refined occupancies are more accurate for high-symmetry unit cells, such as for Nb_0.84_CoSb, than for low-symmetric unit cells. Palatinus and co-workers previously compared the Fe/Mg occupancy in (Mg_*x*_Fe_1−*x*_)_2_Si_2_O_6_ refined from the Bragg reflections in 3DED data with the Fe/Mg occupancy refined from the Bragg reflections in single-crystal X-ray diffraction data, and found a difference in Fe/Mg occupancy of 0.028 (8) (Palatinus, Corrêa *et al.*, 2015 ▸).

For the single-crystal X-ray diffraction data, the *R* value improved significantly after refinement of the displacements of Sb and Co atoms (Roth *et al.*, 2021 ▸), whereas for the 3DED data, the *R* value stayed approximately the same (Table 1 ▸). The average displacements refined from the Bragg reflections in 3DED data [0.175 (6) Å for Sb and 0.180 (8) Å for Co] were calculated using equation (3) and differ by 0.040 (5) Å from the average displacements refined from the Bragg reflections in single-crystal X-ray diffraction data [0.141 (1) Å for Sb and 0.130 (1) Å for Co]. Palatinus and co-workers previously compared the atomic positions refined from the Bragg reflections in 3DED data with the displacements refined from the Bragg reflections in single-crystal X-ray diffraction data for three different samples and found a difference in bond length between 0.01 and 0.02 Å (Palatinus, Corrêa *et al.*, 2015 ▸). The difference in bond length is thus larger for Nb_0.84_CoSb than for the samples reported by Palatinus, Corrêa *et al.* (2015 ▸), which is probably due to differences in the crystal mosaicity. The atomic numbers of the elements of the samples in Palatinus, Corrêa *et al.* (2015 ▸) are lower than the atomic numbers of Nb, Sb and Co. Because the average mean free path between two scattering events is smaller in samples with elements with higher atomic numbers, the intensities in the 3DED data acquired on Nb_0.84_CoSb are more influenced by multiple scattering than the intensities in the 3DED data acquired on the samples in Palatinus, Corrêa *et al.* (2015 ▸).

**Figure 2:**
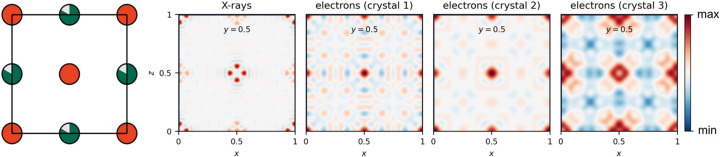
Left: the average positions of Sb (orange) and Nb (green) atoms in the *x*0.5*z* plane. Right: difference Fourier maps in the *x*0.5*z* plane after refinement of the average structure of the thermally quenched sample (Q-0.84 #2) with the centre model, for both single-crystal X-ray diffraction data and 3DED data acquired on three different crystals. The difference Fourier map of the X-ray diffraction data shows differences in the electron density, whereas the difference Fourier maps of the electron diffraction data show differences in the electrostatic potential.

Fig. 2 ▸ shows the difference Fourier maps [ 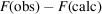 ] in the *x*0.5*z* plane after refinement of the average structure of the thermally quenched sample (Q-0.84 #2) with the centre model, for both the single-crystal X-ray diffraction data and the 3DED data acquired on the three different crystals. The average positions of Sb and Nb atoms in the *x*0.5*z* plane are shown on the left. The red/blue features in the difference Fourier map represent the residual electron density for X-ray diffraction and the residual electrostatic potential for electron diffraction. In the difference Fourier map of the single-crystal X-ray diffraction data, four maxima in the residual electron density can be observed around the average position of the Sb atom, which indicate splitting of the Sb position. Splitting of the Sb position is unclear from the difference Fourier maps of the 3DED data acquired on crystal 1 and crystal 2, but is clear from the difference Fourier map of the 3DED data acquired on crystal 3, even though the maxima in the residual electrostatic potential are broader than for X-ray diffraction. Differences between the difference Fourier maps of the three crystals are likely due to systematic errors in the calculation of the intensities in the dynamical refinement (due to *e.g.* strong multiple scattering, low data-to-parameter ratio, a high crystal mosaicity, no optimization of the frame orientation angles). This also explains the difference between the average Sb displacement refined from the Bragg reflections in 3DED and single-crystal X-ray diffraction data.

### 3.2. Single-crystal electron diffraction versus single-crystal X-ray diffraction

In-zone selected area electron diffraction (SAED) patterns were previously acquired on Nb_0.8_CoSb (Xia *et al.*, 2019 ▸). The main advantage of 3DED is that it allows the acquisition of 3D electron diffuse scattering data with less multiple scattering compared with in-zone SAED patterns.

**Figure 3:**
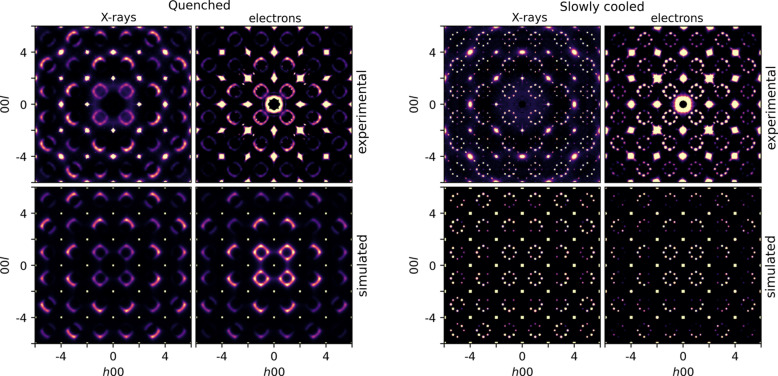
Comparison of the *h*0*l* plane from single-crystal X-ray and single-crystal electron diffraction, for both the thermally quenched sample (Q-0.84 #2) and the slowly cooled sample (SC-0.81). The top row shows the experimental diffuse scattering; the bottom row shows the diffuse scattering calculated in *Scatty* from the structure models calculated in *DISCUS*. The experimental single-crystal X-ray diffraction data were previously reported by Roth *et al.* (2021 ▸).

3DED data were acquired on the thermally quenched sample Nb_0.84_CoSb (Q-0.84 #2) and the slowly cooled sample Nb_0.81_CoSb (SC-0.81). The crystals used for the diffuse scattering analysis were larger than the crystals used for the dynamical refinement in the previous section. Fig. 3 ▸ shows the *h*0*l* plane reconstructed from single-crystal X-ray diffraction and single-crystal electron diffraction data acquired on both samples. The *h*0*l* plane for −20 ≤ *h*,*l* ≤ 20 is shown in Fig. S3. The *h*0.5*l* and *hhl* planes are shown in Figs. S4 and S5.

The diffuse scattering intensity at lower scattering angles is higher for electron diffraction than for X-ray diffraction, which can be explained by differences in the atomic form factors for electrons and X-rays. Fig. S6 shows the X-ray and electron atomic form factors of Co, Nb and Sb as a function of the scattering angle. The X-ray atomic form factor is the Fourier transform of the electron density, whereas the electron atomic form factor is the Fourier transform of the electrostatic potential. Because the electrostatic potential is broader than the electron density, the electron atomic form factors decrease faster to zero than the X-ray atomic form factors, which explains the difference in the intensity distribution of the diffuse scattering.

The bottom row in Fig. 3 ▸ shows the *h*0*l* plane of the 3D diffuse scattering calculated in *Scatty* from the structure models calculated in *DISCUS*. For the thermally quenched sample (Q-0.84 #2), the diffuse scattering was calculated from the short-range order model with a correlation coefficient for nearest-neighbour vacancies of *c*_(1/2,1/2,0)_ = −0.19 and a correlation coefficient for next-nearest neighbour vacancies of *c*_(1,0,0)_ = −0.012. The values for *c*_(1/2,1/2,0)_ and *c*_(1,0,0)_ were determined based on a series of Monte Carlo simulations and gave the best visual agreement between the observed and calculated diffuse scattering. Note that the values for *c*_(1/2,1/2,0)_ and *c*_(1,0,0)_ are different from those used for the calculation of the diffuse scattering by Roth *et al.* (2021 ▸). The diffuse scattering was calculated for an Sb displacement of 0.141 Å and a Co displacement of 0.130 Å (displacements refined from the Bragg reflections in single-crystal X-ray diffraction data in Table 1 ▸). For the slowly cooled sample (SC-0.81), the diffuse scattering was calculated from the long-range order model. Symmetry with the Laue class *m* 3 *m* was applied to the 3D diffuse scattering calculated in *Scatty* (Fig. S7).

### 3.3. Monte Carlo refinement

The Monte Carlo refinement was performed on a model crystal with 25 × 25 × 25 unit cells. The limited crystal size introduces high-frequency noise in the calculated diffuse scattering. The diffuse scattering was averaged over ten crystals to reduce the high-frequency noise. For each crystal, the diffuse scattering was also averaged over 50 lots with a size of 12 × 12 × 12 unit cells, randomly distributed within the model crystal. The lot size should be larger than the correlation length of the longest correlations but smaller than or equal to the crystal size divided by two (Paddison, 2019 ▸). Increasing the crystal size, the number of crystals and the number of lots reduces the noise but increases the refinement time. Figs. S10 and S11 show that increasing the crystal size from 25 × 25 × 25 unit cells to 30 × 30 × 30 unit cells, increasing the number of crystals from 10 to 20, and increasing the number of lots from 50 to 500 improves the quality of the calculated diffuse scattering only marginally.

The Monte Carlo refinement was used to refine the correlations between nearest neighbour vacancies *c*_(1/2,1/2,0)_ and next-nearest neighbour vacancies *c*_(1,0,0)_. Because nearest and next-nearest neighbour vacancies avoid each other, both *c*_(1/2,1/2,0)_ and *c*_(1,0,0)_ should be negative. According to equation (2), the maximum negative value is *c*_(1/2,1/2,0)_ ≃ −4.78 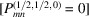 , where 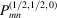 is the probability that two nearest neighbouring Nb sites *m* and *n* are occupied by Nb atoms. However, for Nb_0.84_CoSb a value of 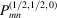 = 0 cannot be achieved and the maximum achievable negative value is *c*_(1/2,1/2,0)_ ≃ −0.20.

The effect of the ratio *c*_(1/2,1/2,0)_/*c*_(1,0,0)_ and the displacements of Sb and Co atoms on the intensity distribution of the diffuse scattering is shown in Fig. S12. Because the intensity distribution of the diffuse scattering depends on the ratio *c*_(1/2,1/2,0)_/*c*_(1,0,0)_, *c*_(1/2,1/2,0)_ was fixed to −0.20 (a value close to the actual correlation coefficient, determined based on a series of Monte Carlo simulations) and *c*_(1,0,0)_ was refined. In total, three parameters were refined: the correlation between next-nearest neighbour vacancies [*c*_(1,0,0)_], the distance between a vacancy *i* and a neighbouring Sb atom *k*(τ_*ik*_), and the distance between a vacancy *i* and a neighbouring Co atom *k*′( 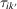 ). Because next-nearest neighbour vacancies avoid each other, starting values for *c*_(1,0,0)_ were chosen in the range [−0.60, −0.01]. The average distance between a vacancy *i* and a neighbouring Sb atom *k* is τ_*ik*_ = 2.801. Because Sb atoms move towards neighbouring vacancies, starting values for τ_*ik*_ were chosen in the range [2.545, 2.945]. The average distance between a vacancy *i* and a neighbouring Co atom *k*′ is 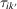 = 2.680. Because Co atoms move away from neighbouring vacancies, starting values for 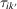 were chosen in the range [2.550, 2.950].

The Monte Carlo refinement was applied to the diffuse scattering in the *h*0*l* plane from the single-crystal X-ray diffraction data and the 3DED data of the thermally quenched sample (Q-0.84 #2) in Fig. 3 ▸. The spatial resolution of the observed diffuse scattering is determined by various effects including the convergence of the beam, the detector point spread function and the crystal mosaicity. To account for resolution effects, the intensity of each pixel in the calculated *h*0*l* plane was convoluted with a Gaussian function (Fig. S13). The standard deviation of the Gaussian function (σ = 0.006 Å^−1^ for X-ray diffraction and σ = 0.008 Å^−1^ for electron diffraction) was estimated from the intensity profile of unsaturated Bragg reflections.

The refinements were interrupted manually after 19 refinement cycles. The refined short-range order parameters are listed in Table 2 ▸. The standard uncertainties of the short-range order parameters are underestimated. Systematic errors could be due to (i) the limited number of correlations included in the model, (ii) non-perfect background subtraction, (iii) inaccurate resolution function correction, (iv) distortions in the reconstructed 3D diffuse scattering (*e.g.* due to small crystal movements during data acquisition or the instability of the goniometer of the TEM sample stage) and (v) no correction for multiple scattering.

In *DISCUS*, there is a difference between target correlations and displacements (the refined correlations and displacements) and achieved correlations and displacements (the actual correlations and displacements) (Neder & Proffen, 2008 ▸). The achieved correlations and displacements were calculated from the final structure model after the refinement. The achieved Co displacements are identical to the target Co displacements, whereas the achieved Sb displacements are lower than the target Sb displacements. The reason is that an Sb atom with two neighbouring vacancies on opposite sides of the Sb atom will not move away from its average position. A Co atom may also have two neighbouring vacancies, but these two neighbouring vacancies cannot be on opposite sides of the Co atom [Fig. 1 ▸(*b*)]. Each Co atom with at least one neighbouring vacancy will thus move away from its average position.

The *R* value in Table 2 ▸ is much higher for the short-range order parameters refined from the diffuse scattering in the 3DED data than for the short-range order parameters refined from the diffuse scattering in the single-crystal X-ray diffraction data. The evolution of the *R* values and the short-range order parameters during the refinement applied to the diffuse scattering in single-crystal X-ray diffraction and 3DED data are shown in Figs. S14 and S15, respectively.

**Figure 4:**
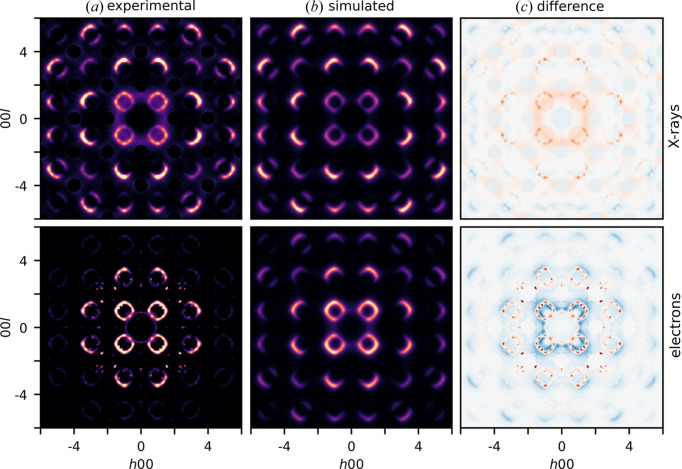
Monte Carlo refinement in *DISCUS* applied to the diffuse scattering in the *h*0*l* plane from the single-crystal X-ray diffraction data and the 3DED data acquired on the thermally quenched sample (Q-0.84 #2). Comparison of (*a*) the experimental diffuse scattering, (*b*) the diffuse scattering calculated for the refined short-range order parameters in Table 2 ▸, and (*c*) the differences between observed and calculated intensities (*I*_obs_ − *I*_calc_).

The diffuse scattering calculated for the refined short-range order parameters is shown in Fig. 4 ▸. At lower scattering angles, the observed diffuse scattering intensities are higher than the calculated diffuse scattering intensities. The satellite reflections are also sharper in the observed diffuse scattering than in the calculated diffuse scattering, especially for the diffuse scattering in the 3DED data.

The displacements refined from the diffuse scattering in single-crystal X-ray diffraction data are 0.142 (11) Å for Sb and 0.112 (8) Å for Co. The displacements refined from the diffuse scattering in 3DED data are 0.142 (23) Å for Sb and 0.071 (21) Å for Co. The standard uncertainties only consider random errors in the intensities of the Bragg reflections and are thus underestimated. The difference between the Sb and Co displacements refined from the diffuse scattering and the Sb and Co displacements refined from the Bragg reflections in single-crystal X-ray diffraction data [0.141 (1) Å for Sb and 0.130 (1) Å for Co] is 0.012 (7) Å for the refinement on the diffuse scattering in single-crystal X-ray diffraction data, and 0.03 (2) Å for the refinement on the diffuse scattering in 3DED data. The local Sb and Co displacements refined from the diffuse scattering are thus close to the average Sb and Co displacements refined from the Bragg reflections in single-crystal X-ray diffraction data, for both X-ray and electron diffraction. As 3DED requires much smaller crystal sizes than single-crystal X-ray diffraction, this opens up the possibility to refine short-range order parameters in materials for which no crystals large enough for single-crystal X-ray diffraction are available. The *R* value is higher for the refinement applied to the diffuse scattering in 3DED data (63.5%) than for the refinement applied to the diffuse scattering in single-crystal X-ray diffraction data (37.2%), which indicates that the correlations refined from the diffuse scattering in single-crystal X-ray diffraction data are likely to be more accurate. The higher *R*value for electron diffraction could be due to artefacts in the subtraction of the experimental background or could be due to residual multiple scattering.

The Monte Carlo refinement of the short-range order parameters took about seven days for 19 refinement cycles. The refinement time is proportional to the number of pixels/voxels in reciprocal space and the number of refined short-range order parameters. Therefore, the refinement was carried out against the diffuse scattering in one 2D plane, and only three parameters were refined. Refining short-range order parameters against the 3D diffuse scattering and refining the correlations between further nearest neighbour vacancies could additionally improve the match between observed and calculated intensities.

### 3.4. The 3D-ΔPDF

The 3D difference pair distribution function (3D-ΔPDF) is the Fourier transform of the 3D diffuse scattering in single-crystal diffraction data (Schaub *et al.*, 2007 ▸) and is often used to determine the origin of the observed diffuse scattering. Recently, Schmidt, Klar *et al.* (2023 ▸) showed that the 3D-ΔPDF can also be reconstructed from the diffuse scattering in 3DED data. The 3D-ΔPDF was obtained by removing the Bragg reflections and Fourier transforming the 3D diffuse scattering/satellite reflections (see Fig. S16). 3D-ΔPDF provides information about correlations between neighbouring atoms that are not represented by the average structure. Positive/negative 3D-ΔPDF values mean that the probability of finding scattering densities separated by the corresponding interatomic vector is higher/lower in the real structure than in the average structure (Weber & Simonov, 2012 ▸). Because X-rays are scattered by the electron cloud, the scattering densities in the X-ray 3D-ΔPDF are electron densities. Electrons are scattered by the atomic nucleus and the electron cloud, and the scattering densities in the electron 3D-ΔPDF are thus atomic charge densities.

**Figure 5:**
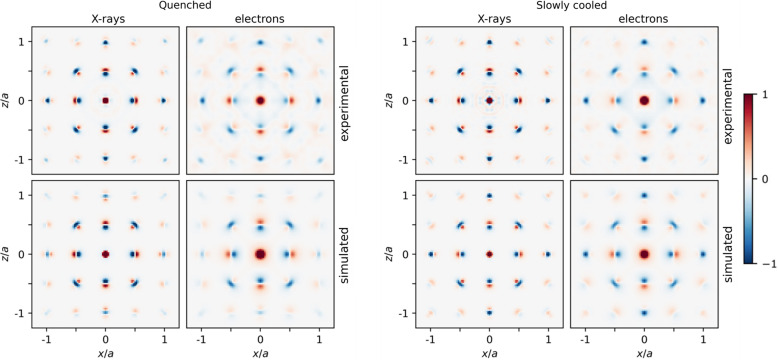
Comparison of the *x*0*z* plane of the X-ray and electron 3D-ΔPDF, for both the thermally quenched sample (Q-0.84 #2) and the slowly cooled sample (SC-0.81). The 3D-ΔPDF was reconstructed from the 3D diffuse scattering data of which the *h*0*l* plane is shown in Fig. 3 ▸. The top row shows the 3D-ΔPDF of the experimental diffuse scattering; the bottom row shows the corresponding 3D-ΔPDF of the calculated diffuse scattering. Positive 3D-ΔPDF features are red and negative features are blue.

Fig. 5 ▸ shows the *x*0*z* plane of the X-ray and electron 3D-ΔPDF, for both the thermally quenched sample (Q-0.84 #2) and the slowly cooled sample (SC-0.81). The 3D-ΔPDF was reconstructed from the 3D diffuse scattering data of which the *h*0*l* plane is shown in Fig. 3 ▸. The calculated 3D-ΔPDF maps are in good agreement with the experimental ones. The displacement of Co atoms can be identified from the *x*0.27*z* plane of the 3D-ΔPDF, for which a similar comparison is shown in Fig. S17. Because the Bragg reflections close to the central beam were overexposed due to the limited dynamical range of the CCD, the Bragg reflections in the electron diffraction data could not entirely be subtracted (Fig. S18). The experimental electron 3D-ΔPDF is thus affected by blooming artefacts due to saturated Bragg reflections (deformation of the features in the 3D-ΔPDF and weak additional features).

The electron 3D-ΔPDF and the X-ray 3D-ΔPDF of Nb_0.84_CoSb contain the same information about correlations between neighbouring atoms, and they can thus both be used to determine the origin of the diffuse scattering. The maximum observable correlation length in the 3D-ΔPDF (8.93 r.l.u.) is determined by the voxel size in reciprocal space (Δ*h*, Δ*k*, Δ*l* ≃ 0.056). The intensity of the diffuse scattering goes faster to zero for electrons than for X-rays (Fig. 3 ▸), which results in broader features in the electron 3D-ΔPDF maps than in the X-ray 3D-ΔPDF maps (Fig. 5 ▸), but which does not hinder the qualitative interpretation of the 3D-ΔPDF. Besides, the diffuse scattering is broader for electron diffraction than for X-ray diffraction, which results in a faster decay of the features in the electron 3D-ΔPDF maps than in the X-ray 3D-ΔPDF maps, as can be seen in Fig. S19.

The 3D-ΔPDF of Nb_0.84_CoSb is similar to the 3D-ΔPDF of Zr_0.82_Y_0.18_O_1.91_ (Schmidt, Neder *et al.*, 2023 ▸; Schmidt, Klar *et al.*, 2023 ▸) and the origin of the diffuse scattering (correlations between neighbouring vacancies and the relaxation of the Zr, Y and O atoms around these vacancies) is also similar. However, the 3D-ΔPDF of Zr_0.82_Y_0.18_O_1.91_ was only interpreted in a qualitative way and no refinement of the short-range order parameters was applied to the diffuse scattering. The features in the 3D-ΔPDF maps of Nb_0.84_CoSb in Fig. 5 ▸ are much sharper than those in the 3D-ΔPDF maps of Zr_0.82_Y_0.18_O_1.91_ (for both electrons and X-rays) (Schmidt, Neder *et al.*, 2023 ▸; Schmidt, Klar *et al.*, 2023 ▸), which can be explained by the different *Q*-range. The 3D diffuse scattering data of Zr_0.82_Y_0.18_O_1.91_ were acquired for −10 ≤ *h*,*k*,*l* ≤ 10, whereas the 3D diffuse scattering data of Nb_0.84_CoSb were acquired for −20 ≤ *h*,*k*,*l* ≤ 20. The *Q*-range is thus higher for Nb_0.84_CoSb (∼21.3 Å^−1^) than for Zr_0.82_Y_0.18_O_1.91_ (∼12.2 Å^−1^). Fig. S20 illustrates that the width of the features in the 3D-ΔPDF is inversely proportional to the *Q*-range.

Short-range order parameters can also be refined from the 3D diffuse scattering using a 3D-ΔPDF refinement in *Yell* (Simonov *et al.*, 2014 ▸). However, at the moment, the 3D-ΔPDF refinement in *Yell* can only be applied to the diffuse scattering in single-crystal X-ray diffraction data. Refining correlations from the diffuse scattering in 3DED data is thus only possible using a Monte Carlo refinement.

## 4. Conclusions

In this article, we have demonstrated the possibility to refine short-range order parameters from the 3D diffuse scattering in 3DED data using a Monte Carlo refinement in *DISCUS*. As 3DED requires much smaller crystal sizes than single-crystal X-ray diffraction, this opens up the possibility to refine short-range order parameters in materials for which no crystals large enough for single-crystal X-ray diffraction are available.

The correlations between neighbouring vacancies and the displacements of Sb and Co atoms were refined from the diffuse scattering in both single-crystal X-ray diffraction and 3DED data acquired on Nb_0.84_CoSb. The local Sb and Co displacements refined from the diffuse scattering in single-crystal X-ray diffraction data [0.142 (11) and 0.112 (8) Å] and 3DED data [0.142 (23) and 0.071 (21) Å] are close to the average Sb and Co displacements refined from the Bragg reflections in single-crystal X-ray diffraction data [0.141 (1) Å and 0.130 (1) Å]. The *R* value was higher for the refinement applied to the diffuse scattering in 3DED data (63.5%) than for the refinement applied to the diffuse scattering in single-crystal X-ray diffraction data (37.2%), which indicates that the correlations refined from the diffuse scattering in single-crystal X-ray diffraction data are likely to be more accurate. The higher *R* value for electron diffraction could be due to artefacts in the subtraction of the experimental background or residual multiple scattering. Since it is not possible to include multiple scattering in the calculation of diffuse scattering intensities, and since the probability for multiple scattering to occur increases with increasing sample thickness, it is important to acquire 3DED data on small crystals.

## 5. Related literature

The following references are cited in the supporting information: Klar *et al.* (2023 ▸); Price *et al.* (2005 ▸); Proffen & Welberry (1998 ▸); Roth *et al.* (2020 ▸); Warren *et al.* (1951 ▸); Welberry & Weber (2016 ▸).

## Supplementary Material

Supporting figures. DOI: 10.1107/S2052252523010254/vq5004sup1.pdf

## Figures and Tables

**Table 1:** Average structure refinement for the thermally quenched sample (Q-0.84 #2) The dynamical refinement from the Bragg reflections in 3DED data acquired on three different crystals is compared with the reference refinement from the Bragg reflections in single-crystal X-ray diffraction data (Roth *et al.*, 2021 ▸). *d*_min_ is the resolution, *N*_tot_ is the total number of reflections used in the refinement, *N*_all_ is the total number of unique reflections used in the refinement, *N*_obs_ is the number of observed unique reflections for which *I*_obs_ > 3σ (*I*_obs_), *N*_outl_ is the number of reflections excluded from the refinement and *N*_par_ is the number of refined parameters. *R*_1_(obs) is the conventional *R* value of the observed reflections, *wR*_2_(all) is the weighted *R* value of all reflections, GOF(obs) is the goodness of fit of the observed reflections and occ_Nb_ is the refined Nb occupancy.

	X-rays	Electrons (crystal 1)	Electrons (crystal 2)	Electrons (crystal 3)
*d*_min_ (Å)	0.40	0.50	0.50	0.45
*N*_tot_	10474	1258	2323	2308

Center model				
*N*_obs_/*N*_all_	202/202	457/457	684/721	717/853
*N*_outl_	0	5	7	5
*N*_par_	7	59	52	56
*R*_1_(obs) (%)	2.76	7.84	11.01	6.62
*wR*_2_(all) (%)	6.00	21.04	27.91	17.81
GOF(obs)	1.318	9.14	9.64	4.92
occ_Nb_	0.831 (1)	0.855 (24)	0.822 (20)	0.798 (12)

Split model				
*N*_obs_/*N*_all_	292/292	459/459	683/720	718/855
*N*_outl_	0	3	8	3
*N*_par_	11	64	57	61
*R*_1_(obs) (%)	0.49	7.85	10.82	6.93
*wR*_2_(all) (%)	0.78	20.92	27.59	18.59
GOF(obs)	0.58	9.12	9.56	5.15
occ_Nb_	0.827 (2)	0.863 (25)	0.811 (20)	0.796 (15)
Sb shift (Å)	0.141 (1)	0.133 (15)	0.183 (8)	0.181 (11)
Co shift (Å)	0.130 (1)	0.184 (8)	0.088 (165)	0.148 (24)

**Table 2:** Monte Carlo refinement in *DISCUS* applied to the diffuse scattering in the *h*0*l* plane from the single-crystal X-ray diffraction data and the 3DED data acquired on the thermally quenched sample (Q-0.84 #2) Refined short-range order parameters and *R* value after 19 refinement cycles. After the refinement, the achieved correlations and displacements were calculated from the target correlations and displacements. *c*_(1/2, 1/2, 0)_ is the correlation between nearest neighbour vacancies and *c*_(1, 0, 0)_ is the correlation between next-nearest neighbour vacancies.

	X-rays		Electrons	
Parameter	Target	Achieved	Target	Achieved
*c*_(1/2, 1/2, 0)_	−0.200	−0.188	−0.200	−0.185
*c*_(1, 0, 0)_	−0.053 (17)	−0.047 (17)	−0.082 (22)	−0.067 (22)
Sb shift (Å)	0.160 (11)	0.142 (11)	0.158 (23)	0.142 (23)
Co shift (Å)	0.112 (8)	0.112 (8)	0.071 (21)	0.071 (21)
*R* value (%)	37.2		63.5	
