# Supplementary material for: Refining short-range order parameters from the three-dimensional diffuse scattering in single-crystal electron diffraction data
Source: IUCrJ. 2024 Jan 1;11(Pt 1):82–91. doi: 10.1107/S2052252523010254 (PMC10833392; doi:10.1107/S2052252523010254)
Supplement: Supplementary file 1 [file m-11-00082-sup1.pdf]

IUCrJ

Volume 11 (2024)

Supporting information for article:

Refining short-range order parameters from the three-dimensional diffuse scattering in single-crystal electron diffraction data

Romy Poppe, Nikolaj Roth, Reinhard B. Neder, Lukas Palatinus, Bo Brummerstedt Iversen and Joke Hadermann

S1. Dynamical refinement of the average crystal structure

Since the intensities of the Bragg reflections in three-dimensional electron diffraction (3D ED) data depend on the crystal thickness, the dynamical refinement requires the refinement of a thickness parameter. The number of refined parameters in the dynamical refinement from 3D ED is much larger than for the kinematical refinement from single-crystal X-ray diffraction. For the kinematical refinement applied to single-crystal X-ray diffraction data, one scale factor was refined for the whole dataset. For the dynamical refinement applied to 3D ED data, one scale factor was refined for each virtual frame, which is necessary because several factors (changes in the illuminated area of the crystal, slowly growing contamination of the crystal, and varying crystal thickness and thus absorption) influence the overall scale of each frame (Palatinus, Corrêa *et al.*, 2015).

The meaning of the dynamical refinement parameters is given below:

$g_{\max}$ is the maximum resolution of reflections used in the dynamical refinement (Klar *et al.*, 2023).

$S_g^{\max}$(matrix) is the maximum excitation error of reflections included in the structure matrix. Determines the number of excited beams entering the structure matrix (i.e., a square matrix with the number of rows equal to the number of excited beams considered in the calculation of the diffracted intensities). The default value for $S_g^{\max}$(matrix) is 0.01 Å^{-1} (Palatinus, Petříček *et al.*, 2015; Palatinus, Corrêa *et al.*, 2015). For materials with heavy atoms, such as $Nb_{0.84}CoSb$, a value of 0.025 Å^{-1} is recommended.

$S_g^{\max}$(refine) is the maximum excitation error of reflections included in the dynamical refinement (Palatinus, Petříček *et al.*, 2015; Palatinus, Corrêa *et al.*, 2015). The default value for $S_g^{\max}$(refine) is 0.1 Å^{-1}.

$R_{S_g}^{\max}$ is the maximum $R_{S_g} = |S_g|/S_g^{\max}$ of reflections used in the dynamical refinement. With S_g the excitation error of a reflection relative to the average goniometer position of the respective overlapping virtual frame. Optimal values of $R_{S_g}^{\max}$ are typically between 0.5 and 0.8 (Klar *et al.*, 2023). Values larger than 0.8 lead to an increase of the R-values. When the number of reflections is lower than ten times the number of refined parameters, the value of $R_{S_g}^{max}$ should be increased (Palatinus, Corrêa *et al.*, 2015).

N_{int} is the number of integration steps. For each virtual frame, the integrated reflection intensities are calculated for N_{int} orientations (Klar *et al.*, 2023). The default value for N_{int} is 100.

S2. Monte Carlo refinement

S2.1. Long-range order model

For the long-range order model, a cell with a size of 6 × 6 × 6 NbCoSb unit cells (cell parameter a = 5.89864(3) Å, space group $F\bar{4}3m$ (Zeier *et al.*, 2017)) was created, and 1/6 of the Nb atoms were replaced by vacancies to form the B1 structure as defined in (Roth *et al.*, 2020). Each Sb atom was moved by 0.148 Å towards its neighbouring vacancy, and each Co atom was moved by 0.128 Å away from its neighbouring vacancy (displacements refined from the Bragg reflections in the single-crystal X-ray diffraction data of the slowly cooled sample $Nb_{0.81}CoSb$ (SC-0.81)) (Roth *et al.*, 2021). The Sb atoms were moved along the cubic <100> directions, while the Co atoms were moved along the cubic <111> directions (Fig. 1(b)). The resulting B1 cell (Fig. S1) has cell parameter a = 35.3918(2) Å and space group *P1*. A structure with a size of 6×6×6 B1 cells was created in *DISCUS*.

In (Roth *et al.*, 2021), the BD structure was used instead of the B1 structure. The BD structure is a combination of the B1 structure and the A2 structure. The BD, B1 and A2 structures are defined in (Roth *et al.*, 2020). Because we noticed that the diffraction patterns calculated from the B1 structure agree better with the experimental diffraction patterns than the ones calculated from the BD structure, we used the B1 structure instead of the BD structure.

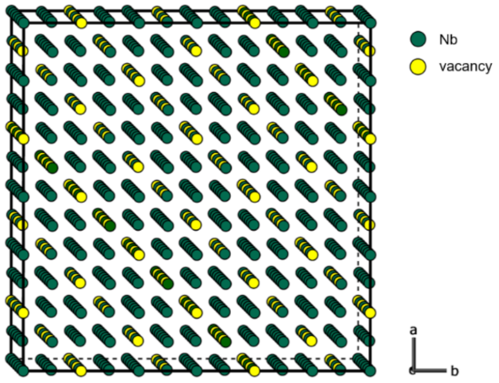

Figure S1 B1 structure, as defined by (Roth *et al.*, 2020), showing the Nb-vacancy ordering in the long-range order model. Sb and Co atoms are omitted for clarity.

S2.2. Short-range order model

For the short-range order model, a starting structure with a size of 25 × 25 × 25 NbCoSb unit cells (cell parameter a = 5.89864(3) Å, space group $F\bar{4}3m$ (Zeier *et al.*, 2017)) was created. 1/6 of the Nb atoms were randomly selected and replaced by vacancies. Periodic boundary conditions were imposed to avoid edge effects.

A Monte Carlo simulation in *DISCUS* is used to minimize the energy *E* of the crystal until the target correlations between nearest neighbour vacancy pairs ($c_{(1/2,1/2,0)}$) and next-nearest neighbour vacancy pairs ($c_{(1,0,0)}$) are achieved. The energy *E* of the crystal is defined as (Roth *et al.*, 2020):

$$E = \frac{1}{2}\sum_{i=1}^{N_{\mathrm{vac}}}\left(J_1\sum_{j}^{12\mathrm{NN}} S_{ij} + J_2\sum_{j'}^{6\mathrm{NNN}} S_{ij'}\right), \quad (1)$$

with N_{vac} the number of vacancies in the crystal. The summation in the first term is over all 12 nearest neighbour (NN) vacancy sites *j* of vacancy *i*, whereas the summation in the second term is over all six next-nearest neighbour (NNN) vacancy sites *j'* of vacancy *i*. $S_{ij} = 1$ if site *j* is occupied by a vacancy and $S_{ij} = 0$ if site *j* is occupied by a Nb atom. Similarly, $S_{ij'} = 1$ if site *j'* is occupied by a vacancy and $S_{ij'} = 0$ if site *j'* is occupied by a Nb atom. J_1 is the energy assigned to a nearest neighbour vacancy pair, and J_2 is the energy assigned to a next-nearest neighbour vacancy pair. Nearest and next-nearest neighbour vacancies will avoid each other when $J_1 > 0$ and $J_2 > 0$. During the Monte Carlo simulation, the values of the energies J_1 and J_2 in Equation 1 are adjusted and the target correlations $c_{(1/2,1/2,0)}$ and $c_{(1,0,0)}$ are calculated. When the target correlations $c_{(1/2,1/2,0)}$ and $c_{(1,0,0)}$ are achieved, the energy *E* of the crystal will achieve its minimum (Neder & Proffen, 2008).

Each Monte Carlo step, two randomly selected Nb atoms/vacancies are switched positions. When the new configuration has a lower energy *E*, then it is always accepted. When the new configuration has a higher energy *E*, then it is only accepted when the transition probability *P*, given by

$$P = \frac{\exp(-\Delta E/kT)}{1 + \exp(-\Delta E/kT)}, \quad (2)$$

is less than a random number η, chosen uniformly in the range [0,1]. ΔE is the energy difference between the new and the old configuration, *T* is the temperature, and *k* is Boltzmann's constant. The temperature *T* controls the proportion of accepted modifications that lead to a higher energy *E*. If *T* = 0, only changes that decrease the energy *E* will be accepted. The higher the temperature *T*, the more moves will be accepted that lead to a higher energy *E* (Proffen & Welberry, 1998; Welberry & Weber, 2016; Neder & Proffen, 2008). In all Monte Carlo simulations, *kT* was chosen equal to 0.001. One Monte Carlo cycle is defined as the number of Monte Carlo steps necessary to visit every crystal site once on average. The number of Monte Carlo cycles was chosen equal to 500 times the number of atoms within the crystal.

A second Monte Carlo simulation in *DISCUS* is used minimize the energy *E* of the crystal until the target distances between a vacancy *i* and a neighbouring Sb atom *k* (τ_{ik}) and between a vacancy *i* and a neighbouring Co atom *k'* ($\tau_{ik'}$) are achieved. The energy *E* of the crystal is defined by a Lennard-Jones potential energy:

$$E = \frac{1}{2}\sum_{i=1}^{N_{\mathrm{vac}}}\left\{\sum_{k}^{6\ \mathrm{Sb}} D\left[\left(\frac{\tau_{ik}}{d_{ik}}\right)^{12} - \left(\frac{\tau_{ik}}{d_{ik}}\right)^{6}\right] + \sum_{k'}^{4\ \mathrm{Co}} D\left[\left(\frac{\tau_{ik'}}{d_{ik'}}\right)^{12} - \left(\frac{\tau_{ik'}}{d_{ik'}}\right)^{6}\right]\right\}, \qquad (3)$$

with N_{vac} the number of vacancies in the crystal and the potential depth $D = -100$. The summation in the first term is over all six neighbouring Sb atoms k of vacancy i, whereas the summation in the second term is over all four neighbouring Co atoms k' of vacancy i. d_{ik} is the distance between a vacancy i and a neighbouring Sb atom k. $d_{ik'}$ is the distance between a vacancy i and a neighbouring Co atom k'. During the Monte Carlo simulation, the values of the distances d_{ik} and $d_{ik'}$ in Equation 3 are adjusted. When the target distances τ_{ik} and $\tau_{ik'}$ are achieved, the Lennard-Jones potential energy will achieve its minimum.

Each Monte Carlo step, one Sb atom is moved towards its neighbouring vacancy and one Co atom is moved away from its neighbouring vacancy. When the new configuration has a lower energy *E*, then it is always accepted. When the new configuration has a higher energy *E*, then it is only accepted when the transition probability *P* in Equation 2 is less than a random number η, chosen uniformly in the range [0,1] (Neder & Proffen, 2008). The number of Monte Carlo cycles was chosen equal to 1000 times the number of atoms within the crystal.

The short-range order model in *DISCUS* was calculated for $kT = 0.001$ (Equation 2). If $T = 0$, only changes that decrease the energy *E* of the crystal will be accepted. The higher the temperature *T*, the more moves will be accepted that lead to a higher energy *E*. Fig. S2 shows the diffuse scattering in the *h0l* plane calculated for different values of *kT*. Differences in the sharpness of the diffuse scattering can be explained by differences between the target and the achieved correlation coefficients. The diffuse scattering was calculated for a target correlation between nearest neighbour vacancies of $c_{(1/2,1/2,0)} = -0.20$ and a target correlation between next-nearest neighbour vacancies of $c_{(1,0,0)} = -0.10$. For $kT = 0.001$, the achieved correlations are $c_{(1/2,1/2,0)} = -0.18$ and $c_{(1,0,0)} = -0.09$, while for $kT = 1$, the achieved correlations are $c_{(1/2,1/2,0)} = -0.16$ and $c_{(1,0,0)} = -0.07$. The achieved correlations are thus lower for higher values of *kT*, which explains the differences in the calculated diffuse scattering.

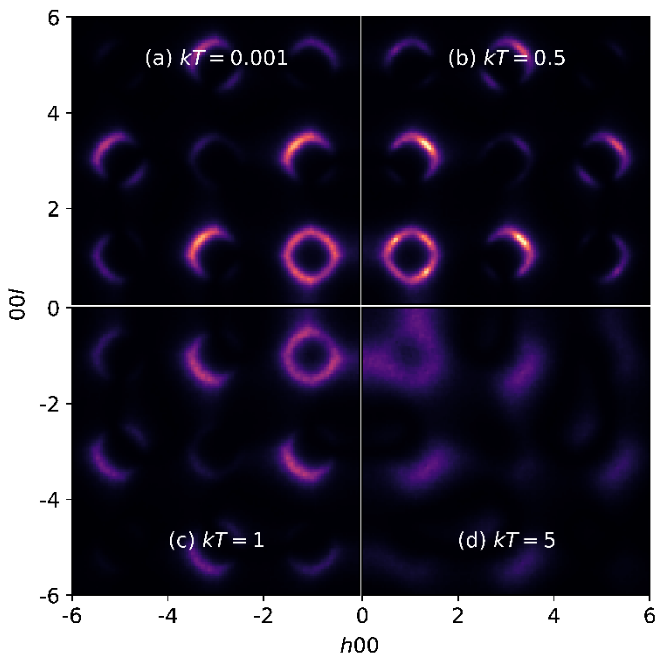

Figure S2 Diffuse scattering in the *h0l* plane calculated for a) *kT* = 0.001, b) *kT* = 0.5, c) *kT* = 1 and d) *kT* = 5. The diffuse scattering was averaged over ten crystals with a size of 25 × 25 × 25 unit cells. For each crystal, the diffuse scattering was also averaged over 50 lots with a size of 12 × 12 × 12 unit cells. The diffuse scattering was calculated for a target correlation between nearest neighbour vacancies of $c_{(1/2,1/2,0)} = -0.20$ and a target correlation between next-nearest neighbour vacancies of $c_{(1,0,0)} = -0.10$.

S2.3. Monte Carlo refinement

A Monte Carlo refinement in *DISCUS* was used to refine the target correlation between next-nearest neighbour vacancy pairs ($c_{(1,0,0)}$), the target distance between a vacancy *i* and a neighbouring Sb atom *k* (τ_{ik}), and the target distance between a vacancy *i* and a neighbouring Co atom *k'* ($\tau_{ik'}$). Each refinement cycle, the short-range order parameters ($c_{(1,0,0)}$, τ_{ik} and $\tau_{ik'}$) are adjusted, and the model crystal is recalculated. The diffuse scattering is calculated and compared with the observed diffuse scattering. This process is repeated until the best agreement between calculated and observed diffuse scattering intensities is obtained.

The differential evolutionary algorithm (Price *et al.*, 2005) mimics the changes in a plant or animal population according to the Darwinian principle of natural evolution. The algorithm starts with a group of M members (parents). Each member represents a set of N short-range order parameters. Next, the algorithm creates a new group of M members (children) by adjusting the short-range order parameters of their parents. The parents and the children with the lowest R-values (Equation 4) survive and will be the parents of the new generation (survival of the fittest). This procedure is

repeated for a number of refinement cycles (generations) until the R-value converges to its minimum (Neder & Proffen, 2008).

$$R_w = \sqrt{\frac{\sum_i w_i [I_{\mathrm{obs}}(\mathbf{Q}_i) - I_{\mathrm{calc}}(\mathbf{Q}_i)]^2}{\sum_i w_i [I_{\mathrm{obs}}(\mathbf{Q}_i)]^2}} \quad (4)$$

The sum is over all measured reciprocal lattice points $\mathbf{Q}_i$, I_{obs} and I_{calc} are the observed and calculated diffuse scattering intensities. The weights w_i were set to unity so that all data points i contribute equally to the summation.

S3. Dynamical refinement of the average crystal structure

Table S1 Average structure refinement for the thermally quenched sample (Q-0.84 #2). The dynamical refinement from the Bragg reflections in three-dimensional electron diffraction (3D ED) data acquired on three different crystals is compared with the reference refinement from the Bragg reflections in single-crystal X-ray diffraction data (Roth *et al.*, 2021). Refined atomic displacement parameters for Sb, Co and Nb (split model in Table 1).

	X-rays	Electrons (crystal 1)	Electrons (crystal 2)	Electrons (crystal 3)
$U_{11,Sb}$ (Å^2)	0.00825(14)	-0.00344(73)	0.00180(231)	0.00357(261)
$U_{22,Sb}$ (Å^2)	0.00495(6)	0.00451(238)	0.00113(127)	0.00562(177)
$U_{33,Sb}$ (Å^2)	0.00495(6)	0.00451(238)	0.00113(127)	0.00562(177)
$U_{12,Sb}$ (Å^2)	0.00000	0.00000	0.00000	0.00000
$U_{13,Sb}$ (Å^2)	0.00000	0.00000	0.00000	0.00000
$U_{23,Sb}$ (Å^2)	-0.00091(2)	0.00106(114)	-0.00023(89)	0.00136(68)
$U_{11,Co}$ (Å^2)	0.00557(9)	-0.00434(144)	0.01062(1075)	0.00729(295)
$U_{22,Co}$ (Å^2)	0.00557(9)	-0.00434(144)	0.01062(1075)	0.00729(295)
$U_{33,Co}$ (Å^2)	0.00557(9)	-0.00434(144)	0.01062(1075)	0.00729(295)
$U_{12,Co}$ (Å^2)	0.00054(6)	-0.00098(69)	0.00348(967)	-0.00039(137)
$U_{13,Co}$ (Å^2)	0.00054(6)	-0.00098(69)	0.00348(967)	-0.00039(137)
$U_{23,Co}$ (Å^2)	0.00054(6)	-0.00098(69)	0.00348(967)	-0.00039(137)
U_{Nb} (Å^2)	0.00479(2)	0.00630(94)	0.00666(100)	0.00886(62)

S4. Single-crystal electron diffraction vs. single-crystal X-ray diffraction

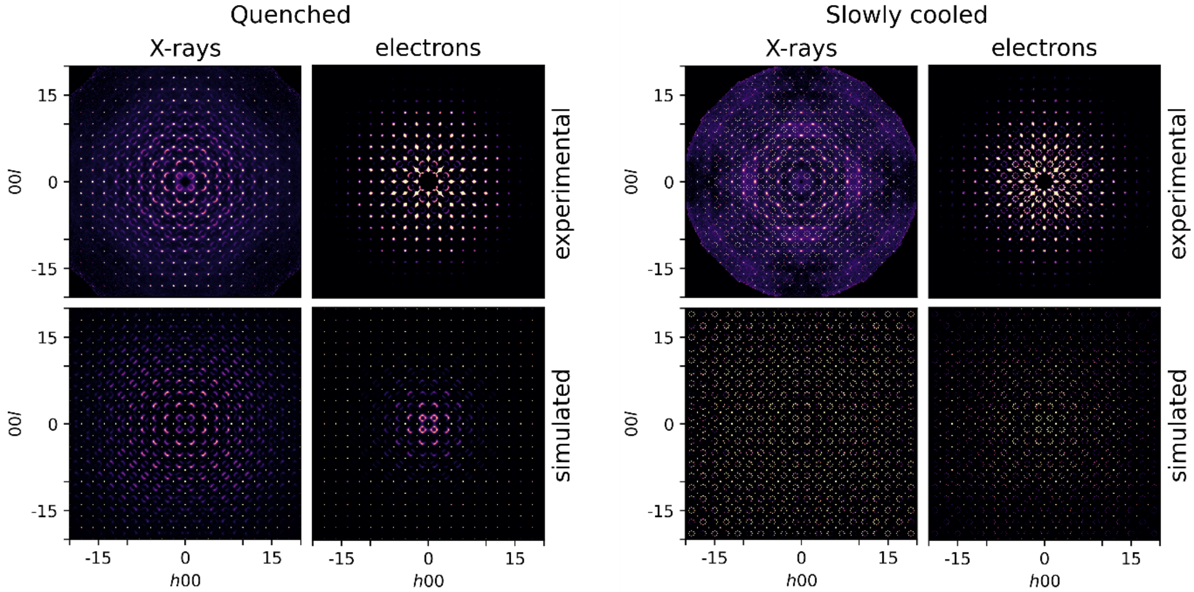

Figure S3 Comparison of the *h0l* plane from single-crystal X-ray and single-crystal electron diffraction, both for the thermally quenched sample (Q-0.84 #2) and the slowly cooled sample (SC-0.81). The top row shows the experimental diffuse scattering; the bottom row shows the diffuse scattering calculated in *Scatty* from the structure models calculated in *DISCUS*. The experimental single-crystal X-ray diffraction data were previously reported by (Roth *et al.*, 2021).

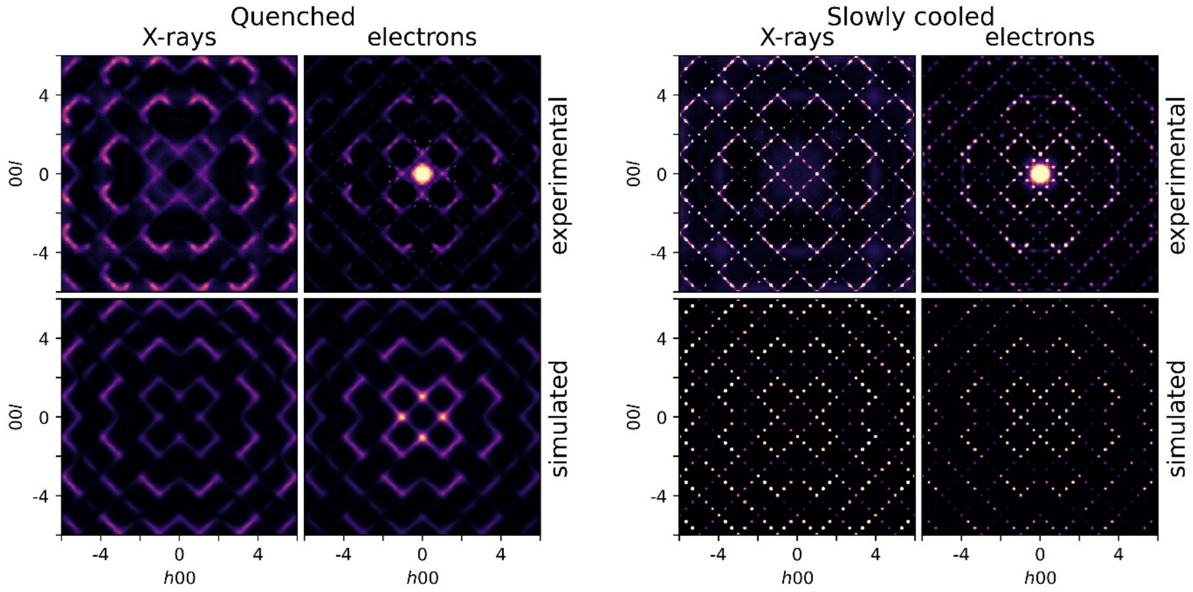

Figure S4 Comparison of the *h0.5l* plane from single-crystal X-ray and single-crystal electron diffraction, both for the thermally quenched sample (Q-0.84 #2) and the slowly cooled sample (SC-

0.81). The top row shows the experimental diffuse scattering; the bottom row shows the diffuse scattering calculated in *Scatty* from the structure models calculated in *DISCUS*.

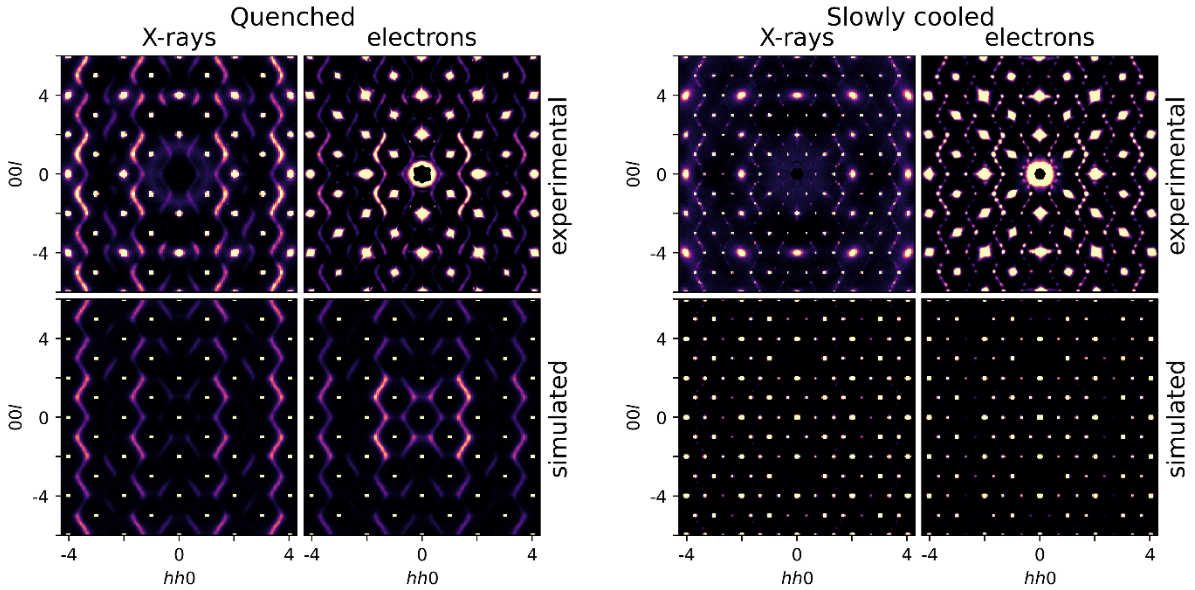

Figure S5 Comparison of the *hhl* plane from single-crystal X-ray and single-crystal electron diffraction, both for the thermally quenched sample (Q-0.84 #2) and the slowly cooled sample (SC-0.81). The top row shows the experimental diffuse scattering; the bottom row shows the diffuse scattering calculated in *Scatty* from the structure models calculated in *DISCUS*. The experimental single-crystal X-ray diffraction data were previously reported by (Roth *et al.*, 2021). Angular broadening of the Bragg reflections is mainly due to crystal mosaicity.

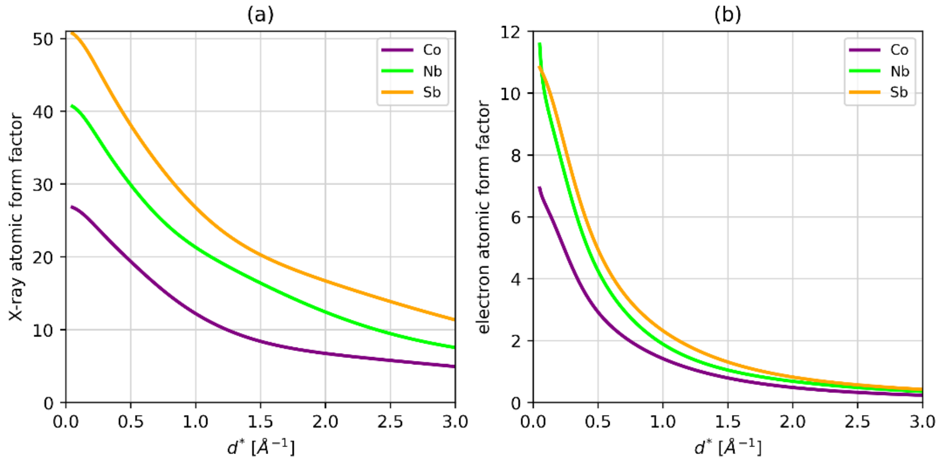

Figure S6 (a) X-ray and (b) electron atomic form factors of Co, Nb and Sb as a function of $d^*=2\sin(\theta)/\lambda$. With d the distance between the lattice planes, θ the scattering angle and λ the wavelength.

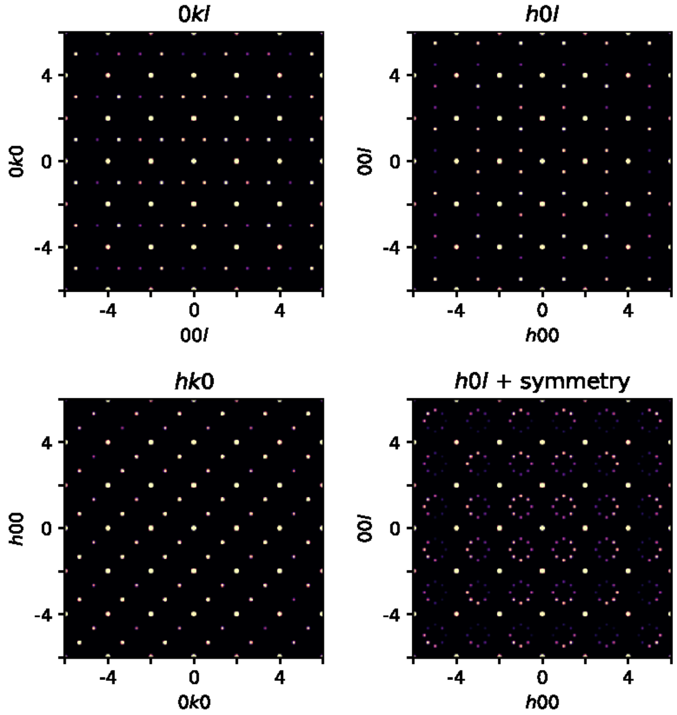

Figure S7 Symmetry with Laue class $m\bar{3}m$ was applied to the three-dimensional diffuse scattering calculated in *Scatty*.

The effect of the vacancy distribution on the *h0l* plane is shown in Fig. S8. The *h0l* plane of a perfectly ordered NbCoSb crystal without vacancies shows sharp Bragg reflections at integer *hkl* values [Fig. S8(a)]. In $Nb_{0.84}CoSb$, 1/6 of the Nb sites are occupied by vacancies. When the vacancy distribution is random and when there are no displacements of Sb and Co atoms, a broad diffuse background will be visible [Fig. S8(b)], which is called monotonic diffuse Laue scattering (Warren *et al.*, 1951). The short-range Nb-vacancy order in Fig. S8(c) results in highly structured diffuse scattering between the Bragg reflections, whereas the long-range Nb-vacancy order in Fig. S8(d) results in sharp satellite reflections.

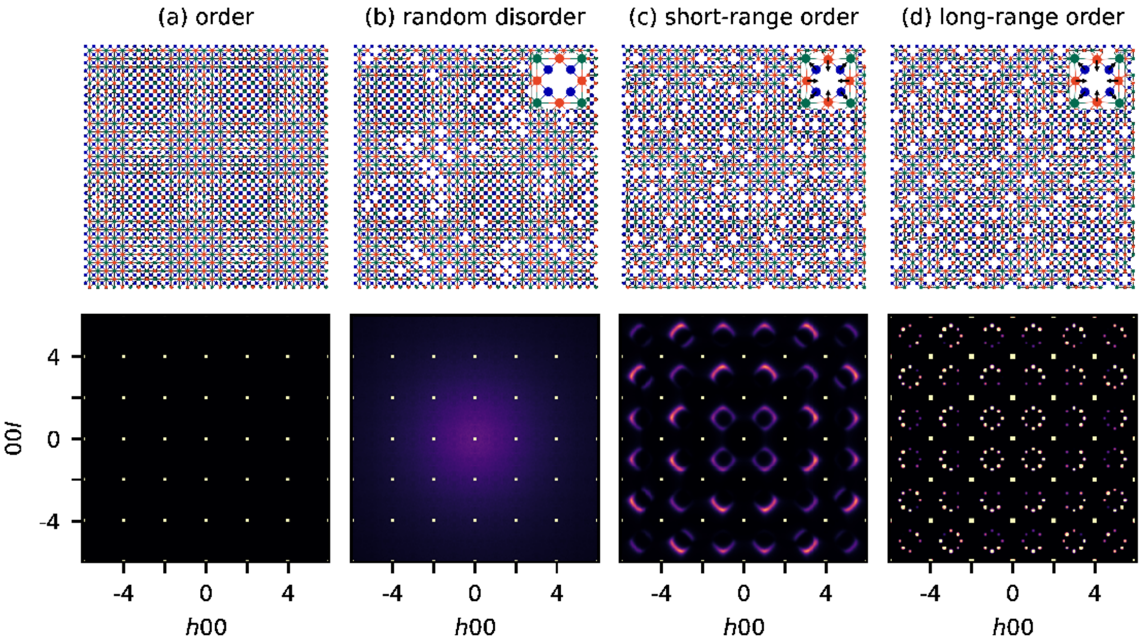

Figure S8 Structure models and their corresponding calculated single crystal X-ray diffraction patterns. (a) The calculated *h0l* plane of a perfectly ordered NbCoSb crystal without vacancies shows sharp Bragg reflections at integer *hkl* values. (b) $Nb_{0.84}CoSb$ structure with a random vacancy distribution and without displacements of Sb and Co atoms. The random vacancy distribution gives rise to monotonic diffuse Laue scattering. (c) $Nb_{0.84}CoSb$ structure with correlations between nearest and next-nearest neighbour vacancies. Displacements of Sb and Co atoms are indicated by arrows. The short-range Nb-vacancy order in (c) results in highly structured diffuse scattering between the Bragg reflections, whereas the long-range Nb-vacancy order in (d) results in sharp satellite reflections.

Fig. S9(a) shows the diffuse scattering calculated for a $Nb_{0.84}CoSb$ crystal with only occupational disorder (correlations between nearest and next-nearest neighbour vacancies). The intensity of the diffuse scattering for a crystal with only occupational disorder decreases with increasing scattering angle. Fig. S9(b) shows the diffuse scattering calculated for a $Nb_{0.84}CoSb$ crystal with only displacive disorder (displacements of Sb and Co atoms around the vacancies). The diffuse scattering for a crystal with only displacive disorder shows asymmetries with respect to the Bragg reflections. It should be noted that the diffuse scattering calculated for the crystal with only displacive disorder looks different from the one reported in the Supporting Information of (Roth *et al.*, 2021), which was calculated using a custom Python script. The observed diffuse scattering in the *h0l* plane in Fig. 3 is thus due to both occupational and displacive disorder (Fig. S9(c)).

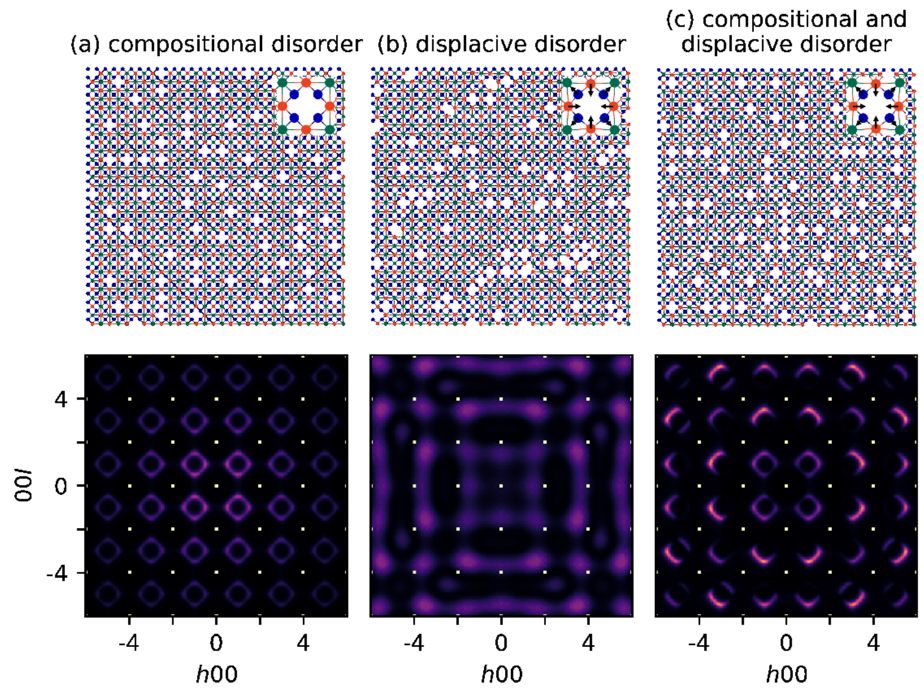

Figure S9 Structure models and their corresponding calculated single crystal X-ray diffraction patterns. (a) The calculated *h0l* plane for a $Nb_{0.84}CoSb$ crystal with only occupational disorder. Correlations between nearest and next-nearest neighbour vacancies give rise to the observed diffuse scattering. (b) The calculated *h0l* plane for a $Nb_{0.84}CoSb$ crystal with only displacive disorder. Displacements of Sb and Co atoms give rise to the observed diffuse scattering. (c) The highly structured diffuse scattering in the *h0l* plane is due to both occupational and displacive disorder.

S5. Monte Carlo refinement

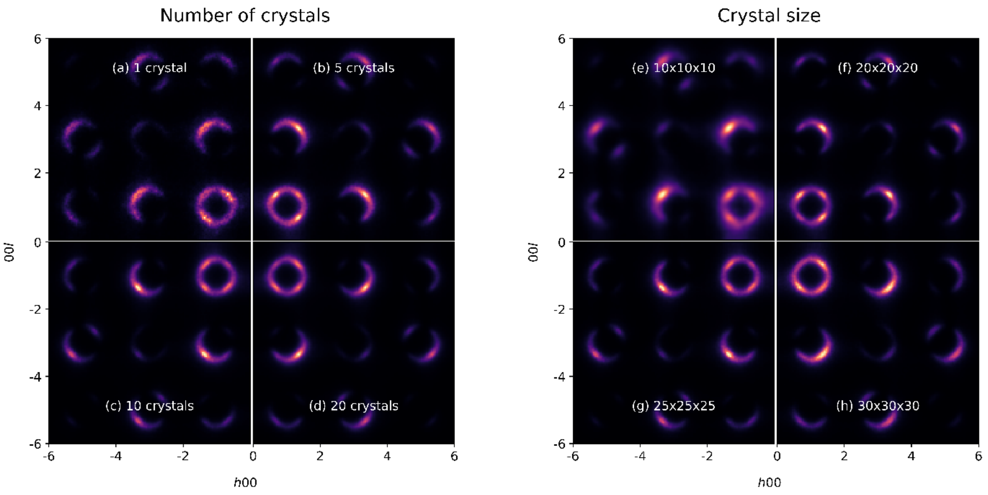

Figure S10 Left: Diffuse scattering in the *h0l* plane averaged over (a) 1 crystal, (b) 5 crystals, (c) 10 crystals, and (d) 20 crystals. For each crystal, the diffuse scattering was averaged over 50 lots with a size of 12x12x12 unit cells. Right: Diffuse scattering in the *h0l* plane averaged over ten crystals with (e) a size of 10x10x10 unit cells and 50 lots with a size of 5x5x5 unit cells, (f) a size of 20x20x20 unit

cells and 50 lots with a size of 10x10x10 unit cells, (g) a size of 25x25x25 unit cells and 50 lots with a size of 12x12x12 unit cells and (h) a size of 30x30x30 unit cells and 50 lots with a size of 15x15x15 unit cells. The lot size should be smaller than or equal to the crystal size divided by two to avoid contributions from periodic images of the crystal. The diffuse scattering was calculated for $c_{(1/2,1/2,0)}/c_{(1,0,0)} = 1$.

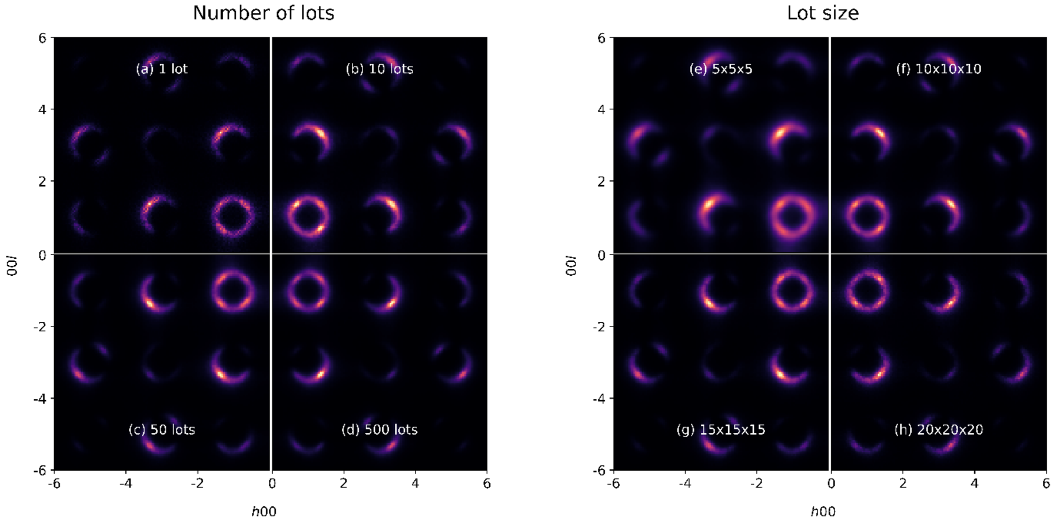

Figure S11 Left: Diffuse scattering in the *h0l* plane averaged over (a) 1 lot, (b) 10 lots, (c) 50 lots, and (d) 500 lots with a size of 12x12x12 unit cells. Right: Diffuse scattering in the *h0l* plane averaged over 50 lots with a size of (e) 5x5x5 unit cells, (f) 10x10x10 unit cells, (g) 15x15x15 unit cells, and (h) 20x20x20 unit cells. The diffuse scattering was calculated for $c_{(1/2,1/2,0)}/c_{(1,0,0)} = 1$ and was averaged over ten crystals with a size of 25x25x25 unit cells.

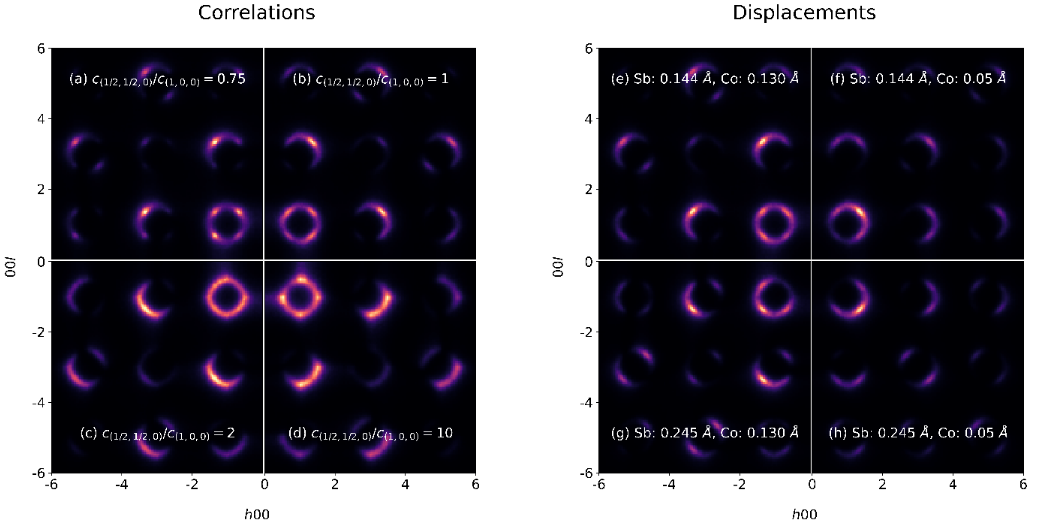

Figure S12 Left: Diffuse scattering in the *h0l* plane calculated for (a) $c_{(1/2,1/2,0)}/c_{(1,0,0)} = 0.75$, (b) $c_{(1/2,1/2,0)}/c_{(1,0,0)} = 1$, (c) $c_{(1/2,1/2,0)}/c_{(1,0,0)} = 2$, and (d) $c_{(1/2,1/2,0)}/c_{(1,0,0)} = 10$. With $c_{(1/2,1/2,0)}$ the correlation between nearest neighbour vacancies and $c_{(1,0,0)}$ the correlation between next-nearest

neighbour vacancies. Right: Diffuse scattering in the *h0l* plane calculated for different displacements of the Sb and Co atoms. The diffuse scattering was calculated for $c_{(1/2,1/2,0)}/c_{(1,0,0)}$ = 1. The diffuse scattering was averaged over ten crystals with a size of 25x25x25 unit cells. For each crystal, the diffuse scattering was also averaged over 50 lots with a size of 12x12x12 unit cells.

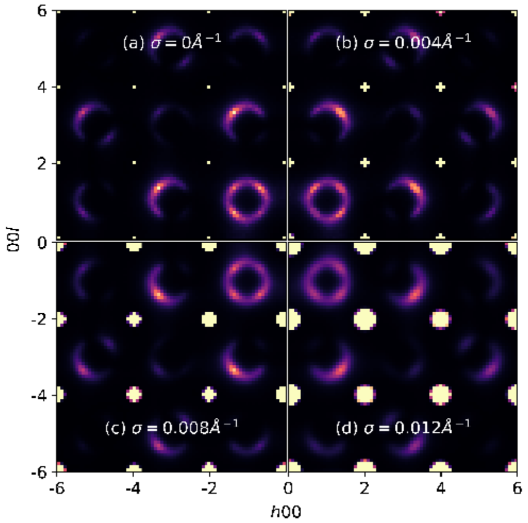

Figure S13 Every pixel in the *h0l* plane was convoluted with a Gaussian with a standard deviation of (a) 0 $Å^{-1}$, (b) 0.004 $Å^{-1}$, (c) 0.008 $Å^{-1}$, and (d) 0.012 $Å^{-1}$. The diffuse scattering was calculated for $c_{(1/2,1/2,0)}/c_{(1,0,0)}$ = 1 and was averaged over ten crystals with a size of 25x25x25 unit cells. For each crystal, the diffuse scattering was also averaged over 50 lots with a size of 12x12x12 unit cells.

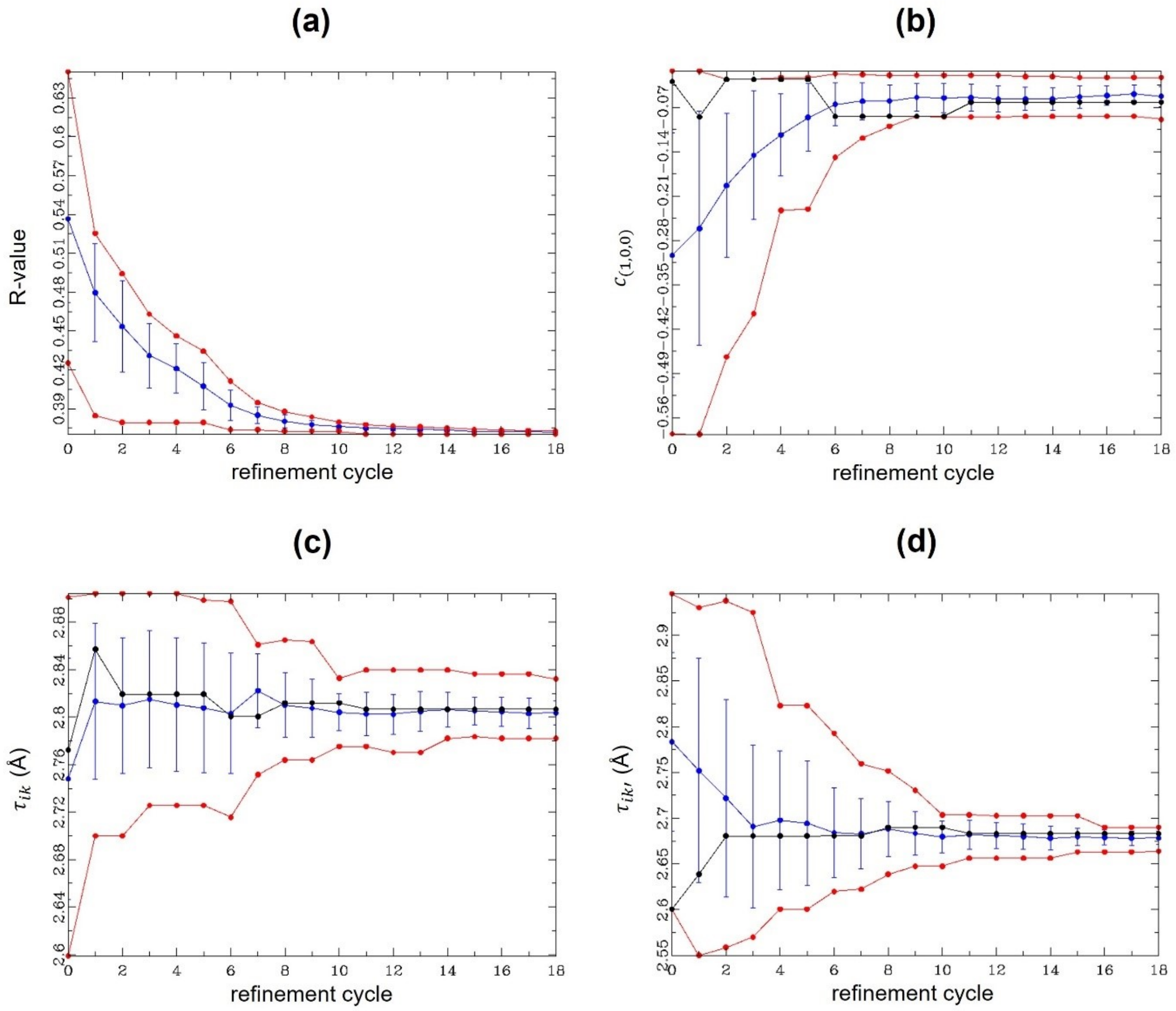

Figure S14 Monte Carlo refinement applied to the diffuse scattering in the *h0l* plane from single-crystal X-ray diffraction data of the thermally quenched sample (Q-0.84 #2). Evolution of (a) the R-value, (b) the target correlation between next-nearest neighbour vacancies ($c_{(1,0,0)}$), (c) the target distance between a vacancy i and a neighbouring Sb atom k (τ_{ik}), and (d) the target distance between a vacancy i and a neighbouring Co atom k' ($\tau_{ik'}$). The figure shows the average value (blue) and the smallest and highest value (red) at each refinement cycle. The value with the lowest R-value at each refinement cycle is shown in black.

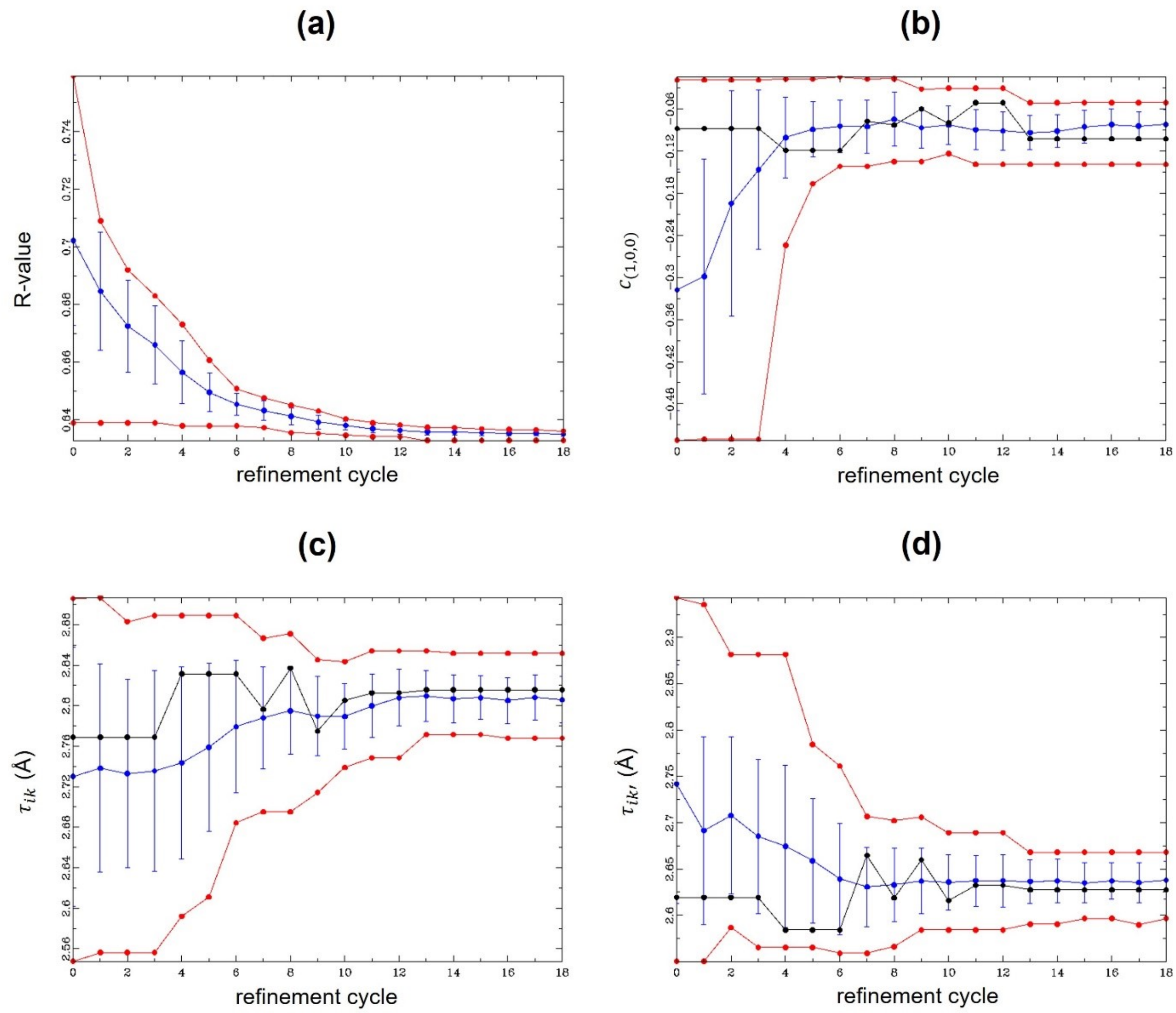

Figure S15 Monte Carlo refinement applied to the diffuse scattering in the *h0l* plane from three-dimensional electron diffraction (3D ED) data of the thermally quenched sample (Q-0.84 #2). Evolution of (a) the R-value, (b) the target correlation between next-nearest neighbour vacancies ($c_{(1,0,0)}$), (c) the target distance between a vacancy i and a neighbouring Sb atom k (τ_{ik}), and (d) the target distance between a vacancy i and a neighbouring Co atom k' ($\tau_{ik'}$). The figure shows the average value (blue) and the smallest and highest value (red) at each refinement cycle. The value with the lowest R-value at each refinement cycle is shown in black.

S6. The 3D-ΔPDF

The *x0z* plane of the experimental 3D-ΔPDF in Fig. 5 is almost identical for the thermally quenched sample (Q-0.84 #2) and the slowly cooled sample (SC-0.81). A positive peak is found at the origin since the distance of an atom to itself is always zero. Strong negative features are visible at interatomic vectors (0.5,0,0.5) and (1,0,0), which correspond to respectively nearest and next-nearest neighbour Nb atoms. The probability of finding two nearest or two next-nearest neighbour Nb atoms is thus lower in the real structure than in the average structure. Consequently, the probability of

finding two nearest or two next-nearest neighbour vacancies is also lower in the real structure than in the average structure. Fig. S19 shows the *x0z* plane of the 3D-ΔPDF for longer interatomic distances. Strong positive features are visible at interatomic vectors (1.5,0,1.5), (2,0,0) and (3,0,0), showing the preferred distances between the vacancies. Besides, the magnitudes of the 3D-ΔPDF features decrease more quickly for the thermally quenched sample than for the slowly cooled sample, which means that the correlation length of the local Nb-vacancy order is longer for the slowly cooled sample than for the thermally quenched sample.

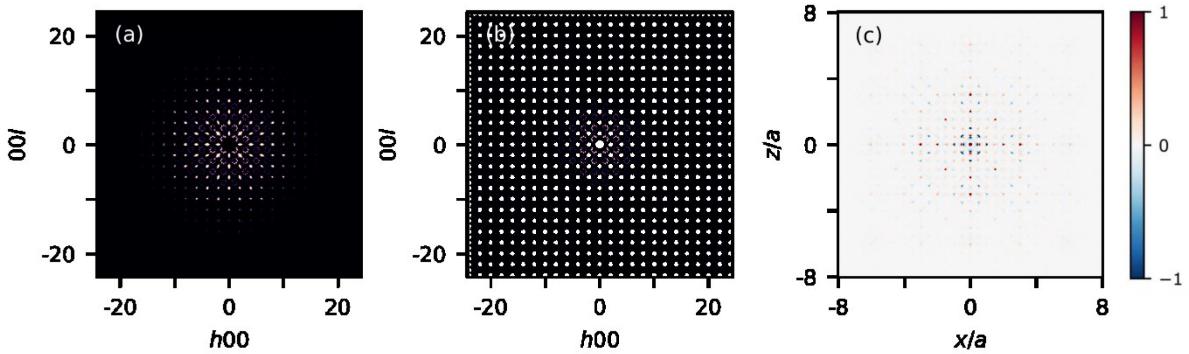

Figure S16 (a) *h0l* plane reconstructed from three-dimensional electron diffraction (3D ED) data acquired on the slowly cooled sample (SC-0.81). (b) *h0l* plane after removing the Bragg reflections. (c) *x0z* plane of the three-dimensional difference pair distribution function (3D-ΔPDF). Positive 3D-ΔPDF features are red and negative features are blue.

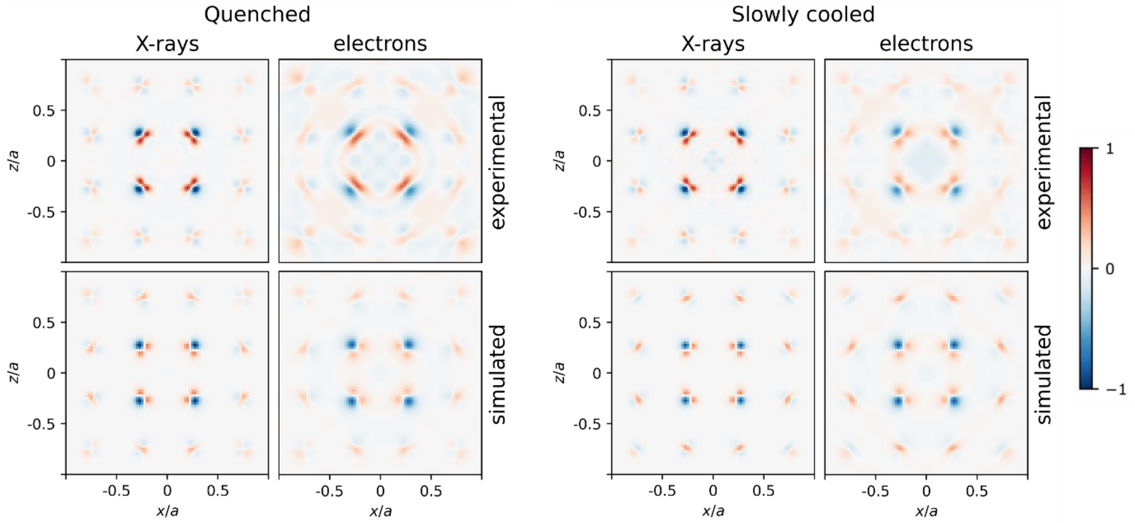

Figure S17 Comparison of the *x0.27z* plane of the X-ray and electron three-dimensional difference pair distribution function (3D-ΔPDF), both for the thermally quenched sample (Q-0.84 #2) and the slowly cooled sample (SC-0.81). The 3D-ΔPDF was reconstructed from the three-dimensional diffuse scattering data of which the *h0l* plane is shown in Fig. 3. The top row shows the 3D-ΔPDF of the

experimental diffuse scattering; the bottom row shows the corresponding 3D-ΔPDF of the calculated diffuse scattering. Positive 3D-ΔPDF features are red and negative features are blue.

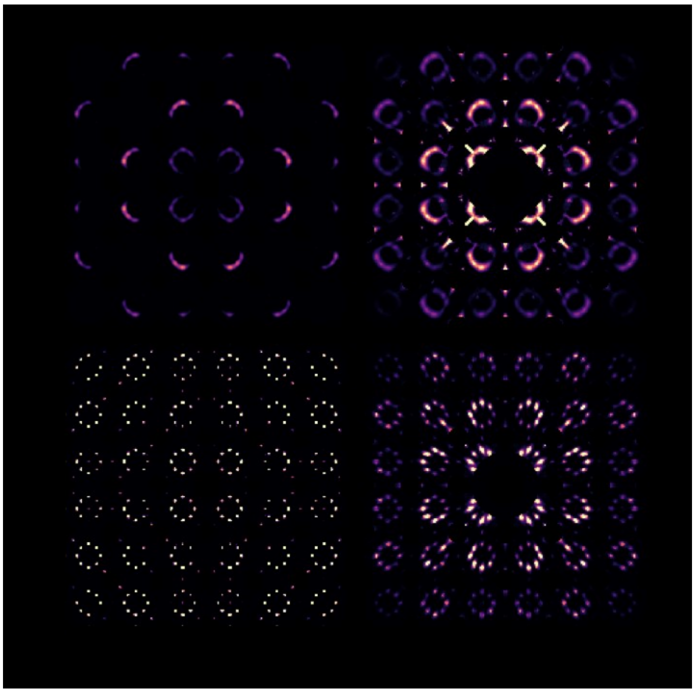

Figure S18 Comparison of the *h0l* plane reconstructed from single-crystal X-ray diffraction and three-dimensional electron diffraction (3D ED) after removing the Bragg reflections, both for the thermally quenched sample (Q-0.84 #2) and the slowly cooled sample (SC-0.81).

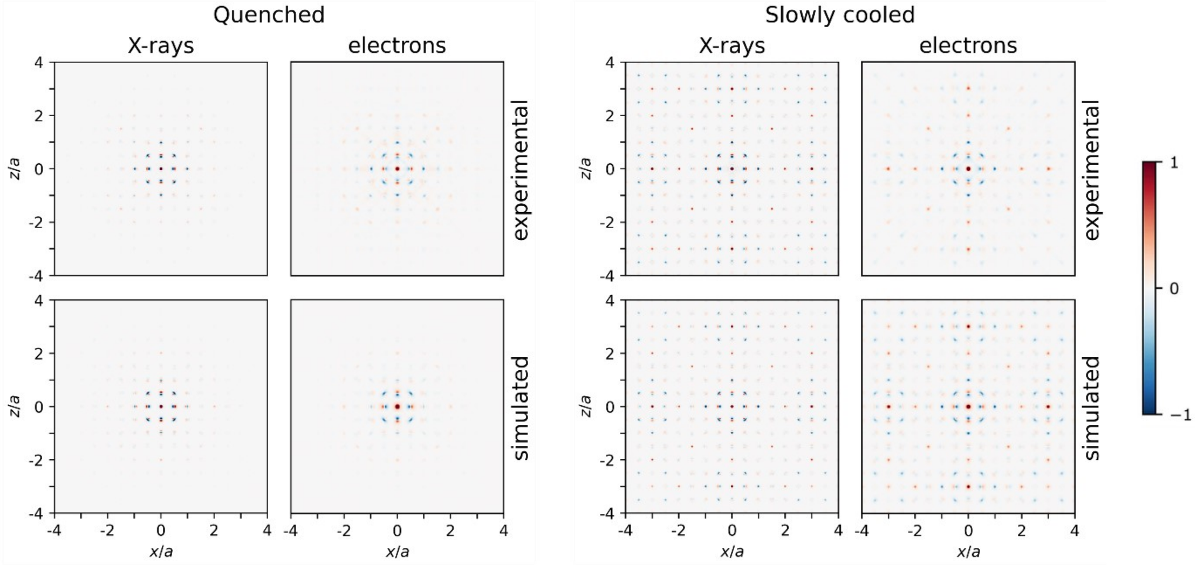

Figure S19 Comparison of the *x0z* plane of the X-ray and electron three-dimensional difference pair distribution function (3D-ΔPDF), both for the thermally quenched sample (Q-0.84 #2) and the slowly cooled sample (SC-0.81). The 3D-ΔPDF was reconstructed from the three-dimensional diffuse scattering data of which the *h0l* plane is shown in Fig. 3. The top row shows the 3D-ΔPDF of the

experimental diffuse scattering; the bottom row shows the corresponding 3D-ΔPDF of the calculated diffuse scattering. Positive 3D-ΔPDF features are red and negative features are blue.

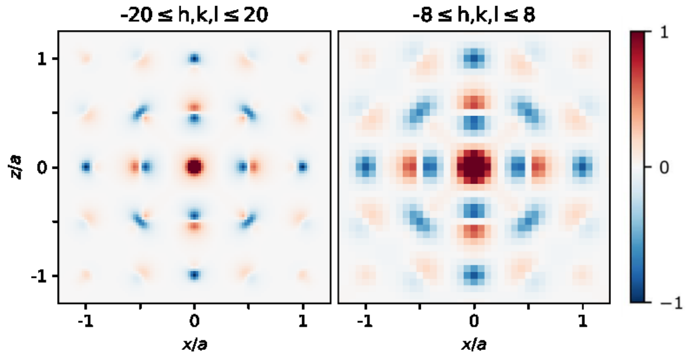

Figure S20 Comparison of the *x0z* plane of the simulated electron three-dimensional difference pair distribution function (3D-ΔPDF) for two different Q-ranges (-8 ≤ *h,k,l* ≤ 8 and -20 ≤ *h,k,l* ≤ 20). The 3D-ΔPDF maps were calculated from the simulated three-dimensional reciprocal lattice of the slowly cooled sample (SC-0.81). Positive 3D-ΔPDF features are red and negative features are blue.
